# Large language models for simplifying radiology reports: a systematic review and meta-analysis of patient, public, and clinician evaluations

**DOI:** 10.1016/j.landig.2025.100960

**Published:** 2026-02-16

**Authors:** Samer Alabed, Abigail Anderson, Ahmed Maiter, Anthony Hughes, Niamh McAnenly, Mahan Salehi, Michael Sharkey, Krit Dwivedi, Alireza Hokmabadi, Fares Alahdab, Mark Stevenson, Ning Ma, Robert Gaizauskas, Tim J Chico, Andy J Swift, Junyi Jessy Li, Jens Kleesiek, Curtis Langlotz

**Affiliations:** a School of Medicine and Population Health, Institute for In Silico Medicine, National Institute for Health and Care Research, University of Sheffield, Sheffield, UK; b Department of Clinical Radiology, Sheffield Teaching Hospitals, Sheffield, UK; c School of Computer Science, University of Sheffield, Sheffield, UK; d Department of Biomedical Informatics, Biostatistics, and Medical Epidemiology and Department of Cardiology, University of Missouri, Columbia, MO, USA; e Department of Linguistics, University of Texas at Austin, Austin, TX, USA; f Institute for AI in Medicine, University Medicine Essen, Essen, Germany; g Department of Radiology, Department of Medicine, and Department of Biomedical Data Science, Stanford University School of Medicine, Stanford, CA, USA

## Abstract

**Background:**

Radiology reports are typically written in language that is difficult for patients to understand. Large language models (LLMs) excel at simplifying text. We aimed to evaluate the ability of LLMs to improve the understanding of radiology reports.

**Methods:**

In this systematic review and meta-analysis, we searched CENTRAL, MEDLINE, and Embase from inception to Nov 11, 2025, without restrictions on language. Full-text articles and preprints were considered for inclusion. Eligible studies applied LLMs to simplify radiology reports and had these reports assessed by members of the public or medical professionals. We excluded studies that focused solely on dialogues with interactive chatbots, preimaging leaflets, educational materials, appointment letters, or summarising findings without simplifying them for patients. Search results were screened independently by two authors and full-text review and data extraction were done by three authors; disagreements were resolved by consensus. The main outcomes were patient, public, and clinician evaluations (Likert scores) and text readability metrics. We assessed study quality with the MAIC-10 tool. This study was registered with PROSPERO (CRD420251027489).

**Findings:**

We identified 2385 records, of which 38 studies were eligible. These 38 studies generated 12 922 simplified reports, assessed by 508 evaluators (387 lay people and 121 clinicians). 35 (92%) of 38 studies used OpenAI GPT models and 29 (76%) produced simplified reports in English. Patients perceived LLM-rewritten reports as significantly more understandable than radiologist reports (mean Likert score 4·04 [SD 1·20] for simplified reports *vs* 2·16 [SD 0·94] for original reports; mean difference 2·00 [95% CI 1·54–2·46]). Clinicians rated LLM-rewritten reports highly for accuracy (mean 4·45 [95% CI 4·27–4·63]; 27 studies) and completeness (mean 4·53 [95% CI 4·30–4·76]; 14 studies). Readability was improved across imaging modalities, with lower Flesch–Kincaid Grade Level for LLM-rewritten reports, including a mean difference of −6·20 (95% CI −6·91 to −5·48) for CT, −5·07 (−5·99 to −4·15) for x-ray, and −5·0 (−6·0 to −4·0) for MRI. The error rate in LLM-rewritten reports was 7·2% (95% CI 5·1%–10·0%; 13 studies) and 0·9% (95% CI 0·6–1·5%; 2 studies) for clinically significant errors.

**Interpretation:**

LLM-simplified radiology reports improved patient-perceived understanding and readability and were rated by clinicians as largely accurate and complete, although a small proportion contained clinically significant errors. LLM-based simplification shows promise for making radiology communication more patient-centred, but further evaluation of its effect on patient outcomes and clinical workflows is required.

**Funding:**

National Institute for Health and Care Research Sheffield Biomedical Research Centre.

## Introduction

When a patient has medical imaging (eg, x-rays, CT, or MRI scans), the images are reviewed by a radiologist who provides a written radiology report. These reports are intended for the requesting clinician and are written in highly technical medical and anatomical detail. Patients are now increasingly accessing and reading such reports.^1^^,^^2^ This access can promote agency, support shared decision making, and reinforce autonomy, reflecting a broader shift towards patient-centred health care, accelerated by legislation and policies. In the USA, the 21st Century Cures Act mandates immediate release of medical records to patients, whereas in the UK and EU, patients have a legal right to access their records under the General Data Protection Regulation. The digital transformation of health-care systems has removed barriers to accessing medical data. For example, patients in the UK can read their radiology reports and other test results on the NHS App.^3^^,^^4^

**Research in context**

**Evidence before this study**

Patients’ access to radiology reports has expanded rapidly, driven by patient-centred care initiatives and policies mandating transparency of medical records. However, most radiology reports are written well above average literacy levels and remain difficult for patients to understand. In parallel, artificial intelligence tools such as large language models (LLMs), trained on vast amounts of text, offer the potential to improve report readability. Despite this promise, evidence from patients, the public, and clinicians is required to determine whether LLM-generated simplifications are comprehensible and accurate. We searched MEDLINE, Embase, CENTRAL, medRxiv, bioRxiv, and arXiv from inception to Nov 11, 2025, with terms related to LLMs, radiology, patient preferences, and reports (appendix p 1). Reference lists were also screened. No restrictions on language or publication type were applied. Eligible studies applied automated methods, including LLMs, to simplify radiology reports and used patient, public, or professional assessors to evaluate comprehension, accuracy, or usability. 38 studies, representing 12 922 simplified reports assessed by 508 raters, were identified, most with small, single-centre samples and variable methods. The meta-analyses suggested that LLM-simplified reports were accurate and complete and improved patient-perceived understanding and readability metrics; however, heterogeneity between studies was high.

**Added value of this study**

This is the first systematic review and meta-analysis to synthesise evaluations of LLM-rewritten radiology reports by both patients and clinicians. Pooled analyses showed that simplified reports were rated substantially more understandable by patients and lay assessors, and clinicians judged them accurate and complete. We also identified limitations, including occasional clinically significant errors, report lengthening, and uncertainties around trust, governance, and workflow integration.

**Implications of all the available evidence**

The combined evidence indicates that LLMs can make radiology reports more accessible to patients without compromising clinical accuracy. However, safe deployment requires human oversight, co-design with patients, and careful integration into workflows. Future research should test LLM-assisted reporting in real-world clinical settings, establish standard evaluation metrics, and explore adaptive formats that balance clarity with brevity. If developed responsibly, LLM-generated simplifications could become a mainstay of patient-centred communication in radiology.

However, because radiology reports are produced by clinicians for clinicians, the technical terminology often poses problems when patients read them. Medical jargon and unfamiliarity with reporting styles can cause confusion,^5^^,^^6^ anxiety,^7^^,^^8^ and reduced satisfaction with care.^9^ Patients misunderstanding radiology reports can promptfurther appointments, investigations, or hospital admissions and therefore can be harmful for both patients and health-care systems.^10^^,^^11^ Furthermore, quality of care could be diminished, as clinician time and attention during an appointment might be diverted away from addressing pertinent medical issues towards explaining minor incidental findings of no relevance.^12^ Enabling access to imaging reports could also perpetuate health-care inequalities. In the UK, 40% of adults struggle with health content, 2% have poor English proficiency, and the average reading age is approximately 9 years.^13^^,^^14^ Therefore, patients with poor literacy or disability might not benefit equally from access to their medical records.

Radiologists and other reporters might have additional concerns. The knowledge that reports will be read by patients could adversely affect the accuracy and quality of reporting. The Royal College of Radiologists notes that radiology departments do not have the capacity to deal with large volumes of queries from patients^15^ and a radiologist might choose to omit findings to reduce queries, even if such findings could be relevant in the future.^6^ Alternatively, a radiologist might be concerned about the emotional effect of their report on a patient and attempt to lessen their description of findings in a way that impairs accurate clinical communication, such as implying cancer by recommending referral to a multidisciplinary team meeting rather than explicitly stating it.

Since patients can access radiology reports, there is a clear need for versions comprehensible to the patient while remaining accurate. In most settings, workload pressure makes it impractical for radiologists to manually produce patient-friendly versions alongside usual clinical reports; rather, automated solutions to this problem are required. Large language models (LLMs) are artificial intelligence (AI) systems that generate human-like text from vast pretraining datasets. In radiology, LLMs are increasingly explored to draft or simplify reports to improve readability in a cost-effective manner.^16^, ^17^, ^18^, ^19^, ^20^, ^21^, ^22^, ^23^ Although more than 1000 AI-enabled radiology tools have US Food and Drug Administration approval, none include generative LLMs for clinical reporting.^24^^,^^25^ LLMs are prone to errors which, although improving, remain a crucial barrier to safe use in high-stakes clinical contexts. These limitations necessitate ongoing clinician validation and raise questions about patient trust and acceptance.^26^, ^27^, ^28^, ^29^

The aim of this study is to evaluate how patients, the public, and medical professionals rate the quality of LLM-rewritten radiology reports. The findings aim to guide the development of patient-centred reporting tools that enhance communication, support equitable health care, and align with the needs and expectations of patients as the ultimate end users of medical imaging services.

## Methods

### Search strategy and selection criteria

In this systematic review and meta-analysis, we searched MEDLINE, Embase, and CENTRAL from database inception to Nov 11, 2025, with the key terms (and their variations) LLM, radiology, patient preferences, and report for full-length articles. The detailed search strategy is provided in the appendix (p 1). In addition, preprint and grey literature articles were searched in medRxiv, bioRxiv, and arXiv with broad terms for radiology and imaging reports (appendix p 1). Reference lists of included studies were also screened. No restrictions on language were applied. Full-text articles and preprints were considered for inclusion.

Studies were eligible for inclusion if they applied automated methods (including LLMs) to rewrite radiology reports (from any imaging modality) to improve patient understanding and used patient, public, or medical assessors to provide qualitative or quantitative assessments of rewritten reports, either with or without subjecting the original medical report to the same assessment. Studies were excluded if they focused solely on dialogues with interactive chatbots, preimaging leaflets, educational materials, appointment letters, or summarising findings without simplifying them for patients. All stages of screening, full-text assessment, data extraction, and quality assessment were done independently, in duplicate, by at least two authors. Search results were screened by two authors (SA and AA) by title, abstract, and keywords. Full-text review was predefined and done by three authors (SA, AA, and NM). Rayyan was used for screening and assessment. Disagreements over inclusion or data extraction were resolved by consensus.

This systematic review and meta-analysis adhered to the PRISMA guidelines (appendix pp 20–21)^30^ and was prospectively registered with the PROSPERO International Prospective Register of Systematic Reviews (CRD420251027489).

### Data analysis

Data extraction was predefined and done independently by three authors (SA, AA, and NM). Duplicate records were checked through Rayyan and Ovid.

Primary outcomes were patient or lay person self-reported understanding of LLM-rewritten radiology reports and clinician-assessed quality of LLM-rewritten reports, both measured with Likert scale scores and summarised as mean differences with 95% CIs. Secondary outcomes were objective readability metrics (eg, Flesch Reading Ease Score [FRES], Flesch–Kincaid Grade Level [FKGL], Automated Readability Index [ARI]), word counts, error rates of LLM-rewritten reports, and comparison of LLM prompting methods. We did sensitivity analyses to evaluate robustness of findings across LLM types (GPT *vs* other LLMs), LLM version (GPT-4 *vs* GPT-3.5), and clinician assessor type (radiologist *vs* non-radiologist).

Extracted information was study characteristics (ie, authors, journal, year of publication, publication type, funding and conflicts of interests, country of origin and language or reports, number of reports, study design, and population details such as demographics, medical history, education level, and language of participants), imaging modality (eg, CT or MRI), body part imaged (eg head or chest), LLM details (eg, dataset, model, prompts, parameters, and hardware used), report assessment methods (eg, type of assessment and number and type of assessors), and outcome metrics (ie, Likert scores, readability scores, and word count). Methodological quality and risk of bias were assessed by two authors (SA and AA) with the MAIC-10 tool.^31^

All meta-analyses were done with R (version 4.4.3) and used a random effects model (metacont function in the meta package for R). Further data analysis details are provided in the appendix (p 2).

### Role of the funding source

The funder of the study had no role in study design, data collection, data analysis, data interpretation, or writing of the report.

## Results

**Figure 1:**
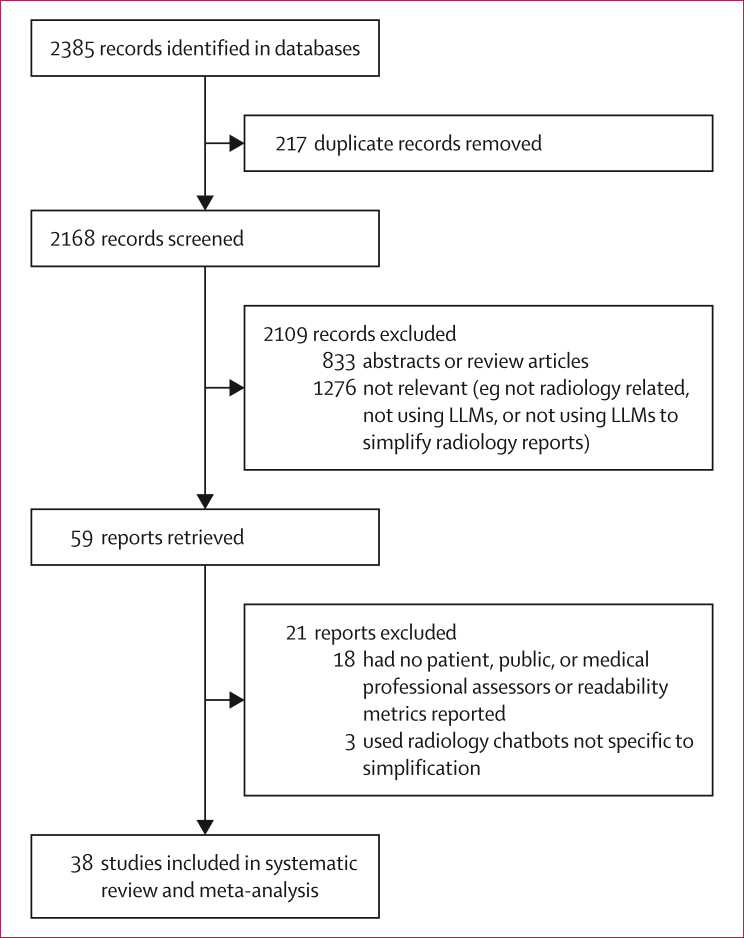
Flow diagram of studies LLM=large language model.

Our search identified 2385 records, of which 38 studies were included (figure 1). These studies were published between 2022 and 2025 and encompassed 12 922 simplified reports evaluated by 508 assessors, of which 387 were lay people (median 17 people [IQR 6–30]) and 121 were medical professionals (median 3 assessors [IQR 2–4]). Study characteristics are presented in table 1.

**Table 1:** Study characteristics table of the included studies

	Language	Imaging modalities included	Radiology subspecialty	Number of reports assessed	Assessors	Large language models used
Amin et al (2023)^32^	English	X-ray, CT, MRI, and ultrasound	Mixed	150	2 medical professionals	GPT 4, Bard, and Copilot
Bai et al (2025)^33^	English	MRI	Mixed	6174	2 medical professionals	GPT-O1 and Deepseek-R1
Berigan et al (2024)^34^	English	X-ray, CT, MRI, ultrasound, and mammography	NR	27	22 lay people	Bard
Berzolla et al (2025)^35^	English	MRI	Musculoskeletal	32	32 lay people and 4 medical professionals	GPT 4
Bozer and Pekçevik (2025)^36^	English	CT and MRI	Mixed	100	2 medical professionals	GPT 3.5, Gemini 2.5 Flash, and Copilot
Butler et al (2025)^37^	English	X-ray, CT, and MRI	Hand musculoskeletal	300	2 medical professionals	GPT 3.5
Butler et al (2024a)^38^	English	X-ray, CT, and MRI	Knee musculoskeletal	300	2 medical professionals	GPT 3.5
Butler et al (2024b)^39^	English	X-ray, CT, and MRI	Foot musculoskeletal	300	2 medical professionals	GPT 3.5
Çamur et al (2024)^40^	Turkish	CT	Mixed	50	3 medical professionals	GPT 4, GPT 3.5, Claude Opus 3, and Gemini 1.5
Can et al (2025)^41^	German	Interventional radiology	Interventional radiology	109	3 medical professionals	GPT 4, GPT 3.5, Claude Opus 3, Gemini 1.5, Mistral 7b, and Mixtral 8 7b
Cesur and Çamur (2024)^42^	Turkish	MRI	Neuroradiology, musculoskeletal, and gastrointestinal	50	3 medical professionals	GPT 4, Claude Opus 3, Gemini, and Perplexity
Chung et al (2023)^43^	English	MRI	Oncology	5	12 medical professionals	GPT 3.5
Doshi et al (2024)^44^	English	X-ray, CT, MRI, ultrasound, and mammography	Mixed	750	NA	GPT 4, GPT 3.5, Bard, and Copilot
Gupta et al (2025)^45^	English	CT	Oncology	50	100 lay people and 3 medical professionals	GPT 4o, Gemini, Claude Opus 3, Llama-3.1 8B, and Phi-3.5-mini
Güneş et al (2024)^46^	Turkish	Ultrasound	Mixed	50	3 medical professionals	GPT 4, Claude Opus, Gemini 1.5, and Perplexity
Jeblick et al (2024)^47^	English	CT and MRI	Neuroradiology, musculoskeletal, and oncology	3	15 medical professionals	GPT 3.5
Kuckelmann et al (2024)^48^	English	MRI	Musculoskeletal	60	3 medical professionals	GPT 4
Li et al (2023)^49^	English	X-ray, CT, MRI, and ultrasound	Mixed	400	NA	GPT 3
Li et al (2025)^50^	English, Spanish, Korean, Chinese, Swahili	CT, ultrasound, and fluoroscopy	Interventional radiology	200	26 lay people and 8 medical professionals	GPT 4
Lyu et al (2023)^51^	English	CT and MRI	Neuroradiology and chest	138	2 medical professionals	GPT 4
Maroncelli et al (2024)^52^	Italian	Mammography, ultrasound, and MRI	Breast	21	5 lay people and 5 medical professionals	GPT 4o
Park et al (2024)^53^	English	MRI	Spinal	685	2 lay people and 2 medical professionals	GPT 3.5
Pisarcik et al (2025)^54^	German	Mammography and ultrasound	Breast	27	40 lay people and 2 medical professionals	GPT 4, GPT 4o, and Gemini 1.5
Prucker et al (2025)^55^	English	CT	Chest	50	3 medical professionals	GPT 4, GPT 3.5, Claude Opus 3, Gemini 1.5, Llama 3 70B, Mistral 7b, and Mixtral 8 7b
Rogasch et al (2023)^56^	English	PET-CT	Oncology	5	3 medical professionals	GPT 4
Salam et al (2024)^57^	English	MRI	Cardiac	20	13 lay people and 2 medical professionals	GPT 4
Sarangi et al (2023)^58^	English, Hindi	CT and MRI	Mixed	9	8 medical professionals	GPT 3.5
Schmidt et al (2024)^59^	German	MRI	Knee musculoskeletal	3	20 lay people and 4 medical professionals	GPT 3.5
Stephan et al (2025)^60^	German	X-Ray	Dentistry	100	300 lay people	GPT 3
Sterling et al (2024)^61^	English	X-ray, CT, MRI, and ultrasound	Mixed	1982	8 medical professionals	GPT 3.5
Sudarshan et al (2024)^62^^,^∗	English	CT, MRI, and ultrasound	Mixed	16	NA	GPT 4o
Sunshine et al (2025)^63^	English	CT and MRI	Neuroradiology	30	4 lay people and 4 medical professionals	GPT 4
Tang et al (2024)^64^	English	CT	Neuroradiology	100	1 medical professionals	Claude 1.3
Tariq et al (2025)^65^	English	CT	Chest	23	3 lay people and 3 medical professionals	T5 fine-tuned
Tepe and Emekli (2024)^66^	English	CT and MRI	Mixed	30	2 medical professionals	GPT 4, Bard, and Copilot
Tripathi et al (2025)^67^	English	X-ray	Chest	500	3 medical professionals	GPT 4
van Driel et al (2025)^68^	Dutch	CT and MRI	Gastrointestinal	10	12 lay people and 2 medical professionals	GPT 4
Yang et al (2024)^69^	English	X-ray	NR	63	8 lay people and 1 medical professional	GPT 3.5

NA=not applicable. NR=not reported.

∗ This paper is a preprint.

Across eight (21%) of 38 studies, 330 patients (median 29 patients [IQR 24–37]) were enrolled either at the time of ordering or attending imaging examinations.^34^^,^^35^^,^^45^^,^^54^^,^^59^^,^^60^^,^^63^^,^^68^ Recruitment was random in one study,^60^ consecutive in three studies,^35^^,^^45^^,^^59^ and based on convenience sampling in four studies.^34^^,^^54^^,^^63^^,^^68^ In addition, two studies^34^^,^^45^ randomly assigned patients to receive either LLM-generated reports or standard reports.

Across six (16%) of 38 studies, 57 lay participants (median 22 participants [IQR 20–53]) were recruited through direct invitation in two studies^57^^,^^69^ and via Amazon Mechanical Turk in one study,^50^ whereas in three studies the recruitment approach was not specified.^52^^,^^53^^,^^65^

The demographics of the patient and public assessors were inconsistently reported (appendix p 3). Eight of 38 studies (21%; 248 participants) reported age data with a pooled mean age of 50 years (SD 18). Sex was reported in eight studies (234 participants), with 131 (56%) participants being female (range 31–100%) and 103 (44%) being male (range 0–69%). Education level was reported in ten studies (26%; 250 participants), and 124 (50%) of 250 were school graduates, 96 (38%) were undergraduates, and 31 (12%) were postgraduates. Ethnicity was reported in only two studies. Four studies did not report any assessors' demographics.

**Figure 2:**
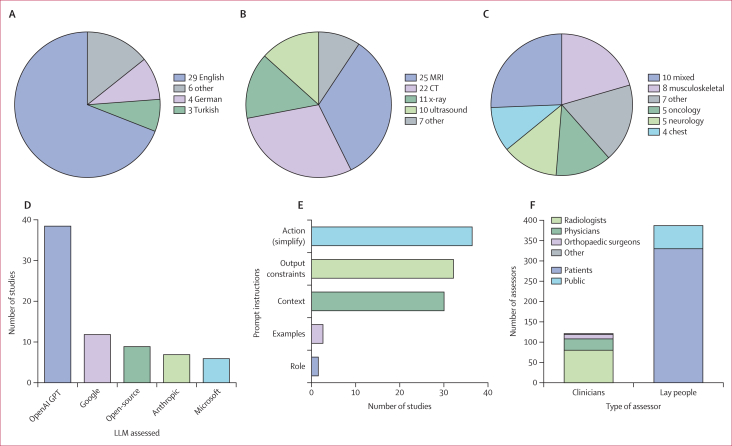
Overview of included studies evaluating LLM-rewritten radiology reports (A) Language of simplified reports across studies (NB, some studies had reports in multiple languages). (B) Imaging modality. (C) Imaging specialty. (D) LLM vendors evaluated. (E) Descriptive information for the prompt anatomy used in report simplification. (F) Assessors of simplified reports. LLM=large language model.

The simplified reports covered six different imaging modalities, with the most common being MRI (25 [66%] of 38) and CT (22 [58%] of 38). Multiple modalities were included in 19 (50%) of 38 studies and multiple imaging specialties in 10 (26%) studies. The most common imaging specialty was musculoskeletal imaging (8 [21%] of 38). Reports were mostly generated in English (29 [76%] of 38). Descriptive information is presented in figure 2.

A single LLM was used in 26 (68%) of 38 studies, whereas 12 (32%) studies compared multiple LLMs. GPT (OpenAI, San Francisco, CA, USA) was used in 35 (92%) studies, with GPT-4 being the most common model (18 [47%] of 38; figure 2).

The included studies reported patient-related and public-related metrics (ie, understanding, trust, empathy, and likability) and medical professional-related metrics (ie, accuracy, completeness, releasability, and harm), most commonly assessed on Likert scales ranging from 1 (strongly disagree or very poor) to 5 (strongly agree or very good).

Quality assessment and the completed MAIC-10 tool for all included studies are available in the appendix (pp 4, 7–8).

**Figure 3:**
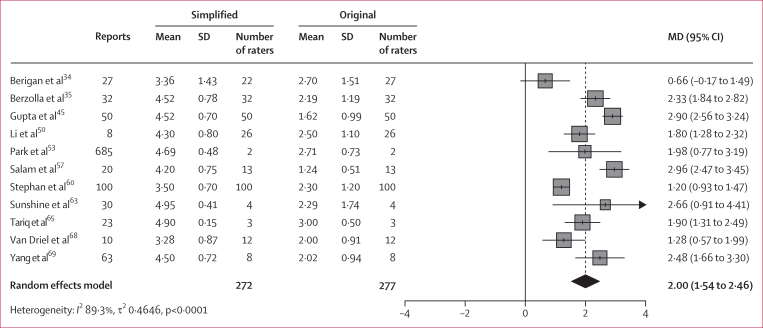
Pooled mean differences in Likert scores for perceived understanding Positive values indicate a preference for simplified reports. MD=mean difference.

The pooled mean Likert score for perceived understanding for original radiologist-generated reports was 2·16 (SD 1·20) across 11 studies (representing 1048 reports and 277 assessors). In comparison, simplified reports generated by LLMs had a pooled mean score of 4·04 (SD 0·94) across 14 studies (representing 1111 reports and 387 assessors). Relative to the original radiologist reports, these data represent an 87% improvement in perceived understanding in patient or lay participant ratings of simplified reports. In studies in which assessors evaluated both the original and LLM-rewritten report, the pooled mean difference (MD) in Likert scores was 2·00 (95% CI 1·54–2·46) across ten studies (representing 1018 reports and 268 assessors), showing that the LLM-rewritten reports were substantially more understandable to lay readers and patients (figure 3).

Patient satisfaction with the radiologist-written report was reported by Sunshine and colleagues,^63^ with a mean score of 1·61 (SD 1·21) across 30 reports and four patients and had an MD of 3·17 (95% CI 1·78–4·56). In comparison, patient satisfaction in LLM-rewritten reports was assessed in three studies^60^^,^^63^^,^^68^ with a pooled Likert score of 3·81 (SD 0·82) across 140 reports and 316 patients. Three studies evaluated how well LLM-rewritten radiology reports conveyed empathy to patients.^54^^,^^55^^,^^60^ The pooled Likert score for empathy was 3·61 (SD 0·79) across 177 reports and 143 patients, indicating a moderately positive perception. No study assessed patients’ perception of empathy in the original radiologist-generated reports.

Patient trust in simplified reports was reported in one study of 32 patients with a mean Likert score of 4·09 (SD 0·68) for the simplified report and 4·48 (0·74) for the radiologist report.^35^

In 32 (84%) of 38 studies, 121 medical evaluators—including 80 (66%) radiologists, 28 (23%) physicians, and 11 (9%) orthopaedic surgeons—participated in the evaluation of the LLM-rewritten reports.

**Figure 4:**
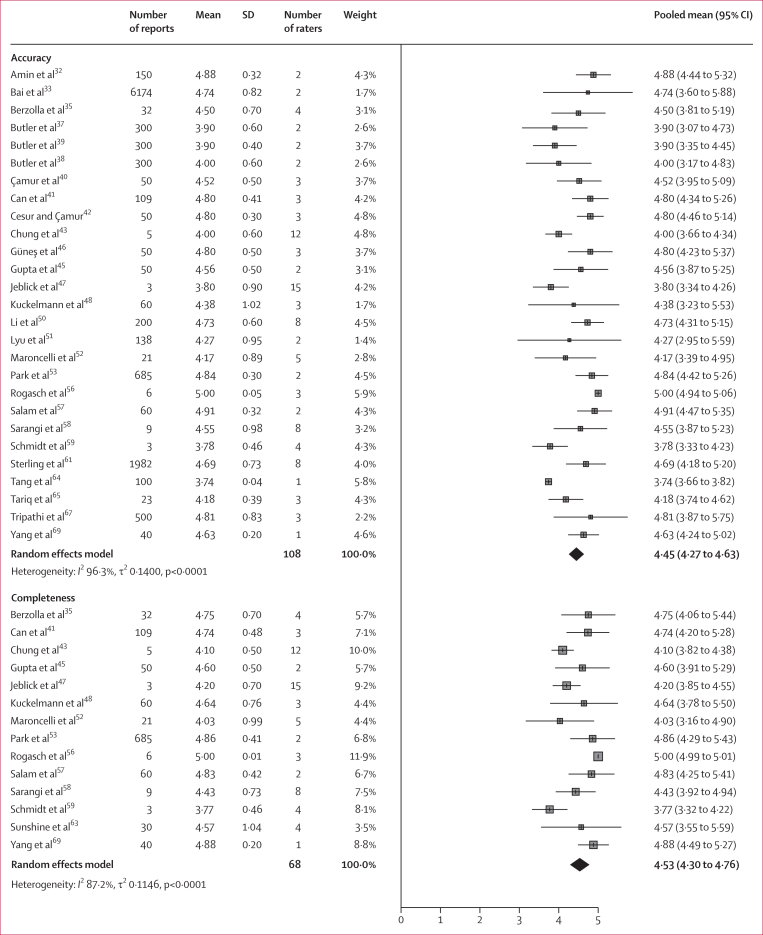

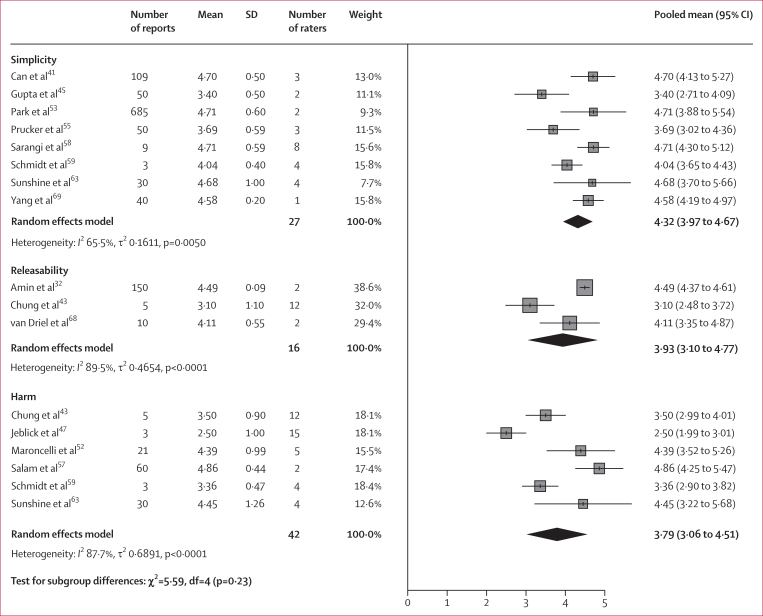
Pooled means in Likert scores for medical professionals’ assessment of large language model simplified reports

Pooled analysis of study outcomes showed high ratings for most measures (figure 4). Accuracy was rated at a pooled mean of 4·45 (95% CI 4·27–4·63) across 27 studies (representing 11 400 reports and 108 raters). Completeness had a pooled mean of 4·53 (95% CI 4·30–4·76) across 14 studies (1113 reports and 68 raters). Simplicity had a pooled mean score of 4·32 (95% CI 3·97–4·67) across eight studies (976 reports and 27 raters). Ratings for suitability to release the LLM-generated reports to patients (ie, the releasability) and for agreement that there was no potential for harm were lower than other scores, with a pooled mean of 3·93 (95% CI 3·10–4·77) for releasability across three studies (165 reports and 16 raters) and 3·79 (95% CI 3·10–4·51) for no potential for harm across six studies (122 reports and 42 raters). The sensitivity analysis restricted to studies using OpenAI models showed that GPT-4 achieved a higher mean accuracy rating of 4·77 (95% CI 4·60–4·94) across 10 studies (1199 reports and 28 raters) than GPT-3.5, with a mean accuracy rating of 4·09 (95% CI 3·78–4·40; p=0·0002) across 12 studies (3786 reports and 62 raters; appendix p 9). Mean accuracy ratings were similar for radiologists (4·51 [95% CI 4·29–4·72] in 17 studies, 1798 reports, and 66 raters) and non-radiologists (4·41 [95% CI 4·07–4·74] in nine studies, 9599 reports, and 38 raters; p=0·62; appendix p 10). Assessor agreement was substantial in six studies and fair or moderate in two studies (appendix p 4).

17 (45%) of 38 studies assessed error rates, including inaccurate, missing, or fabricated information. The pooled error rate for any error was 7·2% (95% CI 5·1%–10·0%) across 13 studies (representing 7774 reports and 353 incorrect reports; appendix p 11). For clinically significant errors, the pooled error rate was 0·9% (95% CI 0·6%–1·5%) across two studies (4037 reports and 38 incorrect reports).

19 (50%) of 38 studies reported readability scores and simplified reports showed substantial improvements across all metrics. A meta-analysis of mean difference in FKGL scores by imaging modality showed consistent improvements in simplified reports across all groups (appendix p 12). For x-ray studies, the pooled MD in FKGL score was −5·07 (95% CI −5·99 to −4·15) across five studies and 1100 reports. For CT studies, the MD was −6·20 (−6·91 to −5·48) across seven studies and 1400 reports, and for MRI, the MD was −5·0 (−6·0 to −4·0) across seven studies and 6729 reports. In studies simplifying multimodality reports, the pooled MD was −4·96 (−6·57 to −3·36) across four studies and 542 reports.

The FKGL, FRES, and ARI readability scores for both the original and simplified x-ray, CT, and MRI reports are summarised in the appendix (p 13). For CT imaging, the original reports mean FKGL score was 13·4 (95% CI 12·4–14·4) and the simplified reports had a score of 7·3 (6·7–7·9), representing a 47% reduction; the original reports mean ARI score was 20·0 (19·4–20·6) and the simplified reports was 9·1 (6·3–12·0), a 57% reduction; and the original reports mean FRES score was 28·0 (25·5–30·5) and the simplified reports were 73·6 (66·7–80·4), a 163% increase. For x-ray reports, the original reports mean FKGL score was 12·4 (11·1-13·6) and the simplified reports score was 7·3 (6·4–8·2), a 42% reduction in FKGL; the original reports mean ARI score was 13·3 (13·2–13·5) and the simplified reports score was 8·3 (3·5–13·0), a 53% reduction in ARI; and the original reports FRES score was 37·1 (35·1–39·1) and the simplified reports score was 77·8 (73·7–82·0), a 111% increase in FRES (appendix p 13). For MRI, the original reports mean FKGL score was 12·5 (11·3–13·8) and the simplified reports score was 7·5 (6·4–8·7), a 21% decrease in FKGL; the original reports mean ARI score was 13·5 (6·8–20·1) and the simplified reports score was 6·1 (3·9–8·2), a 54% decrease in ARI score; and the original reports mean FRES score was 26·3 (21·2–31·4) and the simplified reports score was 67·7 (55·9–79·6), a 72% increase in FRES (appendix p 13). These changes correspond to a shift from university-level to school-level (ages 11–13 years) text and from highly technical language to a general-audience style for CT and x-ray.

Further analyses are found in the appendix on report lengths, LLM model comparisons, and prompting strategies (appendix pp 14–19).

## Discussion

**Figure 5:**
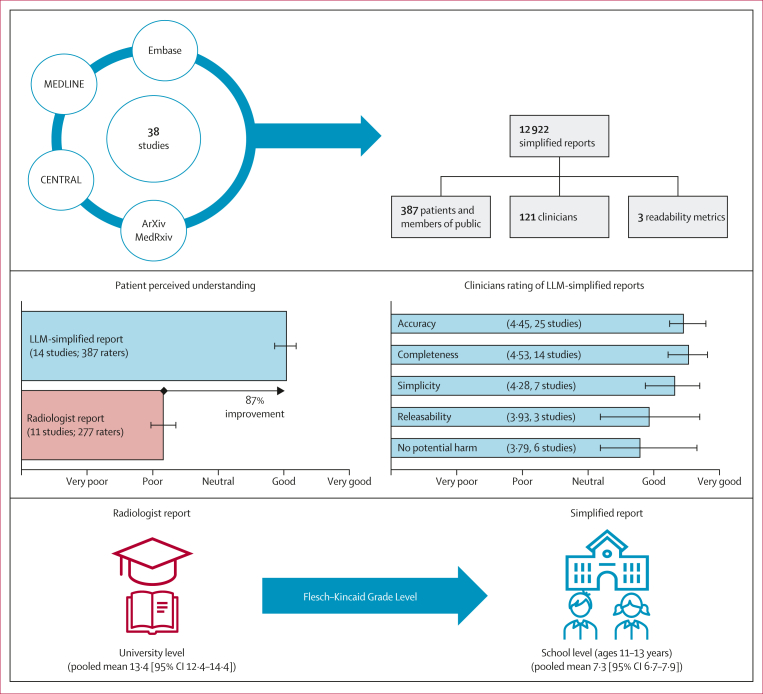
Overview of the findings from this meta-analysis Readability outcomes (Flesch–Kincaid Grade Level) are shown for CT reports. LLM=large language model.

This systematic review and meta-analysis is the first to evaluate LLMs used for improving patient understanding of radiology reports. 38 studies, published since 2022, were included, encompassing multiple imaging modalities, subspecialties, and LLMs. Lay people rated LLM-rewritten reports as 87% more understandable than original radiologist-authored reports. Clinicians also rated them highly for accuracy, completeness, and simplicity. The findings indicate that LLM-simplified reports can improve patient perceived comprehension without sacrificing correctness (figure 5).

Clinicians expressed confidence in the accuracy and completeness of LLM-rewritten reports. Such accuracy is crucial, since legislation such as the 21st Century Cures Act gives patients direct access to radiology reports often written above average literacy levels.^70^ However, this confidence was tempered by caution, consistent with experiences from other LLM-generated explanations.^71^ Releasability and safety were rated lower, reflecting concerns about unsupervised release because of errors, oversimplification, and patient anxiety when accessed without context.

LLM-rewritten reports had a low overall error rate; however, approximately 1 in 100 reports contained clinically significant errors, potentially altering diagnosis or severity. A human-in-the-loop model, in which clinicians review and authorise LLM-generated drafts before patient access, offers a potential solution.^20^^,^^22^^,^^72^ However, the added workload might render this approach impractical in most radiology settings.^73^ The best method for verifying and releasing LLM-reports remains unresolved and no study in this review evaluated real-world use. Responsibility for release also remains unclear. Radiologists are best positioned to check technical accuracy, whereas referring physicians can contextualise results within broader patient care.

The timing of release adds complexity. Instant access risks alarming patients before their clinician has reviewed the findings (eg, reading about fetal death before the clinician can compassionately explain results^74^), whereas delayed access could undermine engagement since patients prefer to know their results before consultations and prepare for discussions with their health-care practitioner.^75^ Governance concerns, liability, disclaimers, and quality assurance remain unresolved, and dissemination methods should ensure equitable access for less digitally literate populations.^76^ Inconsistency in outputs and no standard templates further threaten trust and reproducibility.^20^^,^^77^

Readability remains a central barrier to patient-centred radiology. LLMs consistently improved readability metrics and shifted reports from university-level language to one with a reading age of 11–13 years, aligning with health literacy recommendations.^78^ These improvements should, however, be interpreted with caution. Readability formulas rely on surface-level features of text such as counts of sentences, words, and syllables, which are not consistently defined and vary across tools. Radiology reports in particular are full of abbreviations, numbers, and unusual punctuation, which makes boundary detection difficult and the metrics unstable. Improved readability can also come at the cost of reduced accuracy.^79^^,^^80^ Moreover, traditional readability scores are decades old, can be gamed, and do not necessarily measure comprehension.^81^

Notably, improved readability was often accompanied by longer reports (appendix p 14). This paradox stems from how LLMs simplify: they not only substitute with simpler words but also add definitions, explanations, and analogies to unpack complex terminology. Such expansion might enhance comprehension but also risks information overload and increases burden for clinicians reviewing them. Thus, readability should not be equated with usability.

Although the meta-analysis showed that patients and lay assessors preferred simplified reports, none of the included studies evaluated patients independently applying LLMs to their own radiology reports or explored what patients specifically want to see in these simplified reports. Co-design studies highlighted a shared set of expectations.^82^, ^83^, ^84^, ^85^ Patients prefer reports in clear, lay-friendly language with concise explanations matched to their literacy level. They want unambiguous statements about typical or atypical results, glossaries for technical terms, and structured sections about what the results mean for them and what happens next. Emotional sensitivity is valued when conveying bad news or unexpected findings, alongside actionable advice on urgency, prognosis, and questions to prepare for consultations.^82^, ^83^, ^84^ Visual aids, annotated images, and links to trusted resources help patients' understanding and reduce their anxiety.^82^^,^^83^

Our synthesis found moderately positive experiences with empathy shown in the LLM-rewritten reports. However, trust is more nuanced: in one study, patients rated radiologist reports slightly higher than LLM-rewritten reports for trust,^35^ possibly reflecting patient concerns about depersonalisation, standardised phrasing, bias, or data privacy. Patients supported AI-generated explanations, but only if clinician-supervised to ensure accuracy and transparency.^73^^,^^86^ Patients also want to see both the original and simplified report side by side, for cross-reference and second opinions.^82^^,^^85^ This approach has been adopted in research and commercial solutions, which present the original report with hyperlinks that patients can explore.^34^^,^^87^

The evidence base for the use of LLMs to simplify radiology reports is growing but currently remains small. Most studies were small, single-centre studies. Patient participants tended to be younger, English-speaking, and with higher levels of education, limiting generalisability of the study findings. Full masking of assessors to the report origin is difficult, as LLM outputs have a distinctive style. Studies assessed self-reported (ie, perceived) understanding of radiology reports. However, this perceived understanding might not reflect actual understanding and correlation between the two is not always reliable.^88^ Thus, more accurate ways of assessing comprehension of radiology reports should be investigated, such as tests involving question answering, summarisation, inference, or connection to other knowledge. Reproducibility was constrained by the absence of shared datasets or code and variable LLM prompting methods, which reduces the robustness and transparency of the evidence base. Included studies focused patient and clinician assessments primarily on CT and MRI, limiting generalisability to other imaging modalities. Crucially, no studies incorporated patient perspectives in the design of simplified reports, despite their centrality to patient-centred innovation.

Heterogeneity across the meta-analyses was very high, probably reflecting differences in imaging modalities, clinical specialties, report complexity, disease severity, institutional practices, reporter expertise, assessor experience, rating frameworks, and LLM versions. These differences could not be adjusted for in sensitivity analyses or meta-regression and could reduce the accuracy of pooled estimates. Therefore, the precision and generalisability of pooled effect estimates are reduced, and the summary effects should be interpreted with appropriate caution. Nevertheless, the consistent direction of effect across studies supports the reliability of the overall conclusions.

Future research should prioritise co-design with patients and clinicians and establish standard evaluation metrics and prompting strategies. Prospective implementation studies are needed to assess acceptability, accessibility, equity, safety, workflow effects, and patient-level and system-level outcomes and should incorporate objective comprehension testing (eg, structured questionnaires or assessments) to determine whether improved readability translates into better understanding. To accommodate diverse preferences and literacy levels, future systems might need to offer adaptive formats that balance brevity with detail, such as concise summaries alongside detailed explanations.^70^^,^^89^ Further development should explore embedding visual or explanatory aids to ensure reports are not only more readable but genuinely usable for patients.^90^, ^91^, ^92^

In conclusion, LLM simplification of radiology reports improves patient perceived understanding while maintaining clinical accuracy, positioning it as a powerful tool to make radiology reports more patient centred. By adopting a careful, evidence-based approach, LLM-rewritten reports could evolve from a technical novelty into a cornerstone of patient communication.

## Data sharing

Search terms, study characteristics (data, model, and performance metrics), quality assessment criteria and results, risk of bias assessment criteria and results, and meta-analysis findings are available in the appendix.

## Declaration of interests

We declare no competing interests.
