# Supplementary material for: Large language models for simplifying radiology reports: a systematic review and meta-analysis of patient, public, and clinician evaluations
Source: Lancet Digit Health. 2026 Feb 16;8(2):None. doi: 10.1016/j.landig.2025.100960 (PMC12992207; doi:10.1016/j.landig.2025.100960)
Supplement: Supplementary appendix [file mmc1.pdf]

THE LANCET
Digital Health

Supplementary appendix

This appendix formed part of the original submission and has been peer reviewed. We post it as supplied by the authors.

Supplement to: Alabed S, Anderson A, Maiter A, et al. Large language models for simplifying radiology reports: a systematic review and meta-analysis of patient, public, and clinician evaluations. *Lancet Digit Health* 2026. https://doi.org/10.1016/j.landig.2025.100960

Web appendix to:

Large language models for simplifying radiology reports: a systematic review and meta-analysis of patient, public, and clinician evaluations

Contents

Search strategy 1
OVID - Embase and MEDLINE: 1
medRxiv, bioRxiv, and arXiv: 1

Data analysis 2
Supplementary Table 1: Overview of readability metrics used in the assessment of LLM-rewritten radiology reports. 2
Supplementary Table 2: Patient and public assessor demographics 3

Quality assessment 4
Supplementary Table 3: Summary of reported inter-rater agreement across included studies. 4
Supplementary Figure 1: Funnel plot for the patient/lay understanding outcome. 5
Supplementary Figure 2: Funnel plot for medical professionals' accuracy ratings. 6
Supplementary Table 4: MAIC-10 quality assessment 8

Subgroup analyses 9
Supplementary Figure 3: Forest plot of medical professionals' accuracy ratings stratified by OpenAI GPT-3.5 vs GPT-4. 9
Supplementary Figure 4: Forest plot of medical professionals' accuracy ratings - radiologists vs non-radiologists. 10
Supplementary Figure 5: Forest plot of error rates in LLM-rewritten reports. 11
Supplementary Figure 6: Forest plot of pooled mean differences in Flesch–Kincaid Grade Level (FKGL) scores. 12
Supplementary Table 5: Meta-analytic pooled readability scores for original and LLM-rewritten radiology reports. 13

Report lengths 14
Supplementary Figure 7: Forest plot showing word counts for original and LLM-rewritten reports. 14

LLM comparisons 15
Supplementary Table 6: Comparison of various LLMs with GPT-4 across multiple outcomes. 15
Supplementary Figure 8: Forest plot comparing the performance of LLMs with GPT-4 across multiple outcomes. 16
Supplementary Figure 9: Forest plot comparing LLMs with GPT-4 for FKGL readability scores. 17

Prompting strategies 18
Supplementary Table 7: LLM prompts used in included studies 19

PRISMA checklist 20

References of included studies 22

References of the discussion section 23

Use of ChatGPT in the manuscript 25

Search strategy

OVID - Embase and MEDLINE:

1. exp Artificial Intelligence/
2. exp Natural Language Processing/
3. (“large language” or LLM or ChatGPT or GPT* or Bard or Bert or Claude or Gemini or LLaMA or deepseek or MISTRAL)
4. 1 or 2 or 3
5. exp Radiology/
6. exp Diagnostic Imaging/
7. exp Radiology Information Systems/
8. exp “reporting and data system”/
9. (radiolog* or imaging or MRI or CT or “computed tomography” or “magnetic resonance imaging” or ultrasound or PET)
10. 5 or 6 or 7 or 8 or 9
11. exp Health Literacy/
12. exp Comprehension/
13. exp Patient Education/
14. exp Communication/
15. exp Consumer Health Information/
16. exp Patient Satisfaction/
17. exp Patient Preference/

    (patient adj1 (friendly or preferenc* or cent* or experienc* or satisfaction or perception or readabilit* or literacy or communicat* or comprehens* or understand* or engag* or simpl* or summar* or interpret* or need* or empower*)).mp.
18. 11 or 12 or 13 or 14 or 15 or 16 or 17 or 18
19. (report or text or summar* or finding* or informati* or letter)
20. exp medical record/
21. 19 or 20
22. 4 and 10 and 19 and 21
23. remove duplicates from 22

medRxiv, bioRxiv, and arXiv:

- radiolog* report
- imaging report

Data analysis

Comparisons between original and simplified reports were summarised using descriptive statistics and percentage change. Mean and standard deviation (SD) values for Likert-scale ratings were extracted for all outcome metrics reported by lay or medical professional assessors. When not reported, they were calculated from raw data provided in tables, supplementary materials, or derived from figures using PlotDigitizer.com.

Likert scores for simplified and original reports were pooled in a meta-analysis of mean differences (MD). In five studies, Likert ratings were standardised to create comparable 5-point scales across studies: 0–4 scales were shifted by +1, 10-point scales were linearly rescaled (x 0.5), and 3-point categorical scales were mapped to 1, 3, and 5. Four-point scales (1–4) were linearly rescaled to 1–5 and inverted scales, where 1 indicated highest agreement, were reversed. Transformations were applied only when the questions measured the same type of outcome (e.g., understanding, accuracy) and when the response options followed similar levels of gradations (e.g., from "strongly disagree" to "strongly agree").

Readability metrics (Flesch-Kincaid Grade Level (FKGL), Flesch Reading Ease Score (FRES), and Automated Readability Index (ARI)) were summarised by pooling means and standard deviations by modality for each readability score. For studies reporting multiple modality-specific results (e.g. CT and MR), scores were first averaged within each study to avoid duplication and then aggregated by modality group. FKGL score differences between original and simplified reports were synthesised in a meta-analysis of mean differences. Supplementary Table 1 (page 2) defines each metric.

Robust variance estimation (robumeta package) was applied for LLM model comparisons to address dependence and avoid overweighting multi-outcome studies. Early and later versions of the same models (e.g. BARD and Gemini) were not pooled together, as newer versions offered substantial performance improvements. Because the effect size of the reported error rate was small, a logit transformation of proportions was applied prior to meta-analysis, with pooled estimates back-transformed to proportion scale for presentation. To explore contributors to heterogeneity, we performed sensitivity and subgroup analyses of medical professionals' accuracy assessments, including restricting to OpenAI GPT models (GPT-3.5 vs GPT-4), comparing performance across different LLMs, and stratifying assessors (radiologists vs non-radiologists). Publication bias and small-study effects were evaluated for patient/lay understanding and medical professionals' accuracy using funnel plots and Egger's regression test.

METRIC	YEAR	CALCULATION	GRADING SCALE	USE CASE
**FLESCH READING EASE SCORE (FRES)**	1948	206.835 – 1.015 × (words ÷ sentences) – 84.6 × (syllables ÷ words)	0–100 scale (90–100 = very easy; 0–30 = very difficult)	General readability; often used in journalism, business, and medical texts
**AUTOMATED READABILITY INDEX (ARI)**	1967	4.71 × (characters ÷ words) + 0.5 × (words ÷ sentences) – 21.43	US school grade (rounded to nearest grade level)	Designed for quick computer calculation; educational materials, technical manuals
**FLESCH–KINCAID GRADE LEVEL (FKGL)**	1975	0.39 × (words ÷ sentences) + 11.8 × (syllables ÷ words) – 15.59	US school grade (e.g., score 8 ≈ 8th grade reading level)	Health literacy: indicates years of schooling needed to understand text

Supplementary Table 1: Overview of readability metrics used in the assessment of LLM-rewritten radiology reports.

Author Year	*Feedback*	*N*	*Age*	*Sex F (%)*	*Ethnicity*	*School (%)*	*University (%)*	*Post-graduate (%)*
Berigan 2024	Survey + free text	22	52 ± 14	59	White 95%, Other 5%	9	77	14
Berzolla 2025	Survey	32	53 ± 15	31	White 88%, Other 12%	16	53	31
Gupta 2025	Survey +interview	100	40 ± 13	44	NR	74	20	6
Li 2025	Survey	26	NR	NR	NR	NR	NR	NR
Maroncelli 2024	Survey + interview	5	49 ± 21	100	NR	40	40	20
Park 2024	Survey	2	NR	50	NR	0	100	0
Pisarcik 2025	Survey, ranking and Free text	40	45 ± 14	100	NR	56	44	0
Salam 2024	Survey	13	39 ± 15	54	NR	31	54	15
Schmidt 2024	Survey	20	40 ± 14	55	NR	45	20	35
Stephan 2025	Survey	300	NR	NR	NR	NR	NR	NR
Sunshine 2025	Survey	4	70 ±1 0	NR	NR	25	50	25
Tariq 2025	Survey	3	NR	NR	NR	NR	NR	NR
Van Driel 2025	Survey + Interview	12	66 ± 9	NR	NR	42	58	0
Yang 2024	Survey	8	NR	NR	NR	NR	NR	NR

Supplementary Table 2: Patient and public assessor demographics
N = Number of lay people, F = Female, NR = not reported,

Quality assessment

All studies clearly defined a clinical problem, described their input data, and used appropriate outcome measures and evaluation metrics. In 13/38 (34%) studies, bias was partly mitigated through strategies such as clearing GPT sessions to minimise memory effects, randomisation of report order, blinding of assessors to report origin, consistent grading instructions and ensuring proportional representation of report types and lengths. Model explainability was absent, as almost all studies relied on proprietary LLMs without insight into how outputs were generated. Only Yang (2024) explored transparency through self-correction prompting [69]. Reproducibility was also poor, with no studies making their code or datasets publicly available. One included study was a preprint published in arxiv and was not found in peer-reviewed publications [62]. Medical assessor agreement of medical professionals was reported in 8/29 (28%) studies. Six studies showed substantial agreement between raters. Accuracy was reported fair and moderate in two studies (**Supplementary Table 4**). Visual inspection of the funnel plots **(Supplementary Figures 1 and 2)** and Egger's tests (patient/lay understanding: $t = 0.24$, $df = 9$, $p = 0.82$; medical professionals' accuracy: $t = -0.62$, $df = 24$, $p = 0.54$) provided no evidence of publication bias or small-study effects.

Study	***Metric***	***Reported Values***	***Agreement***
Salam 2024	ICC	Lack of potential harm: 0.93;<br>Completeness: 0.76;<br>Accuracy: 0.55	Almost perfect<br>Substantial<br>Moderate
Sarangi 2023	ICC	0.87	Substantial
Tepe 2024	ICC	0.82	Substantial
Can 2024	Cohen's κ	0.77 - 0.84	Substantial
Kuckelman 2023	Cohen's κ	Accuracy: 0.33;<br>Completeness: 0.29	Fair<br>Fair
Park 2024	Weighted κ	L-Spine 0.86;<br>C-Spine 0.88;	Substantial<br>Substantial
Prucker 2025	Cohen's κ	0.76–0.86	Substantial
Tripathi 2024	% agreement	86 - 95%	Almost perfect

Supplementary Table 3: Summary of reported inter-rater agreement across included studies.
Reported reliability metrics and corresponding interpretation of agreement strength are presented.
ICC = intraclass correlation coefficient; κ = kappa

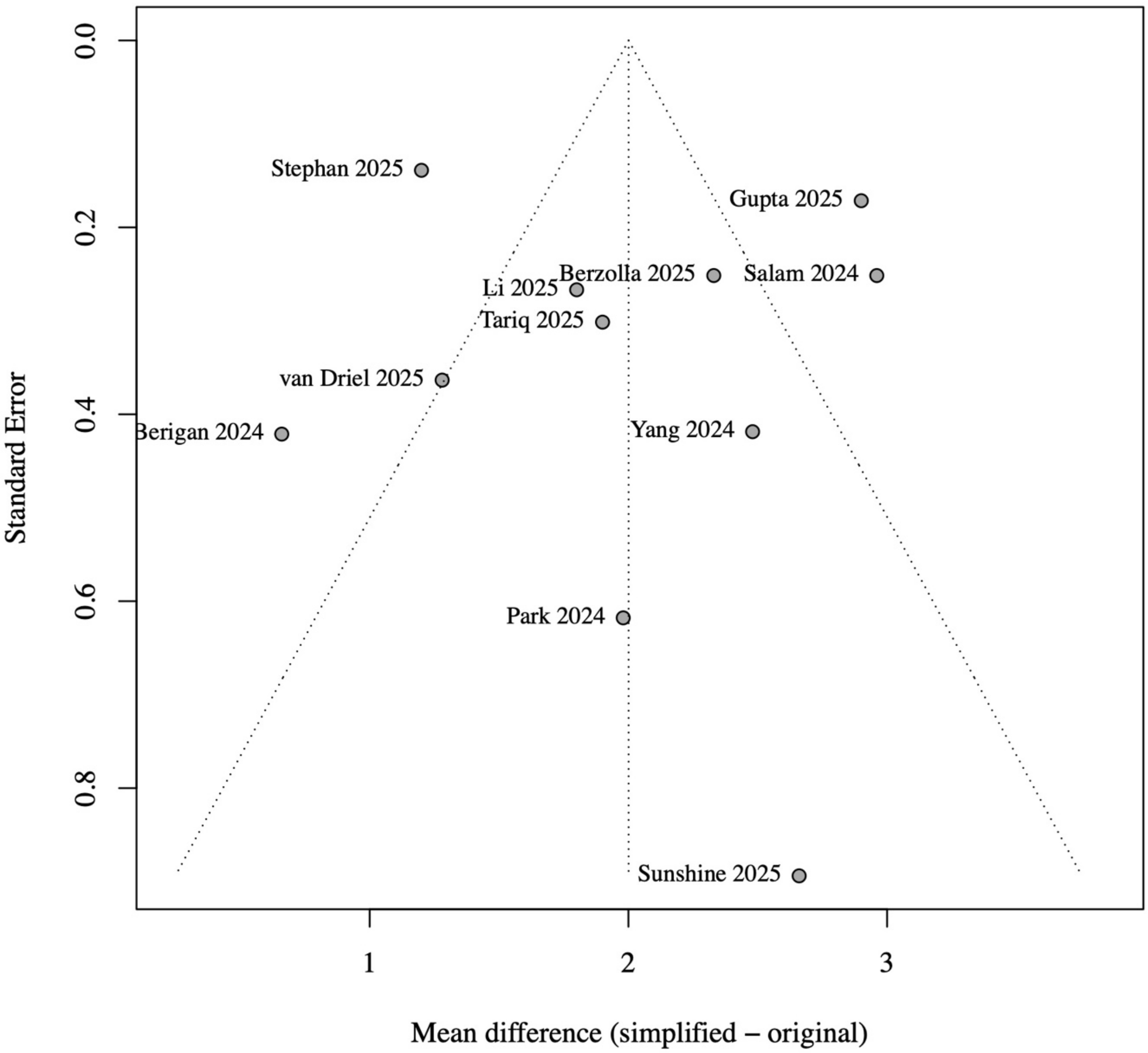

Supplementary Figure 1: Funnel plot for the patient/lay understanding outcome.

Funnel Plot assessing potential publication bias and small-study effects for the patient/lay understanding outcome. The distribution of study effect sizes shows no appreciable asymmetry, supported by a non-significant Egger's test ($t = 0.24$, $df = 9$, $p = 0.82$).

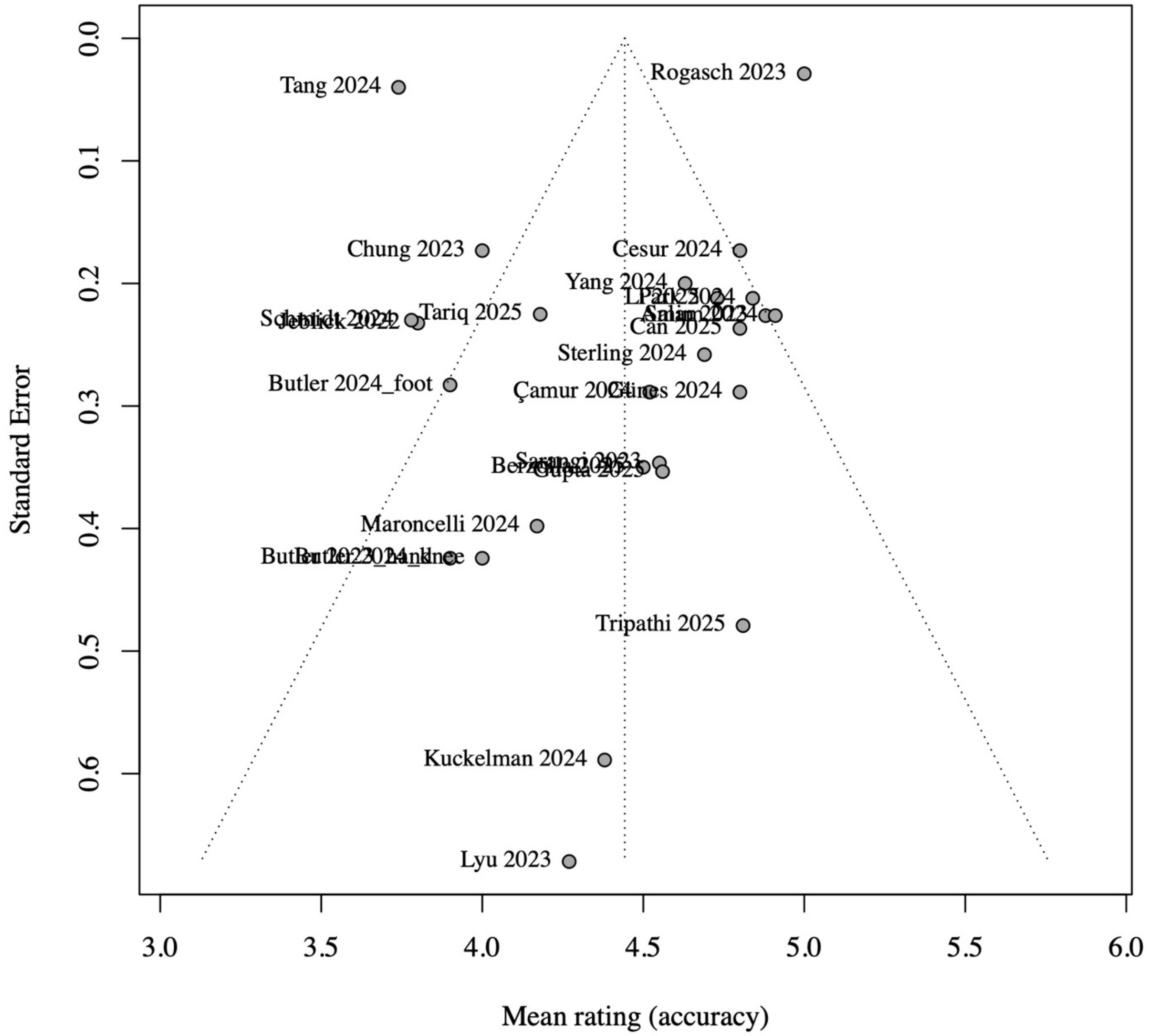

Supplementary Figure 2: Funnel plot for medical professionals' accuracy ratings.

Funnel plot evaluating potential publication bias and small-study effects for medical professionals' accuracy ratings. The plot shows no evidence of asymmetry, consistent with a non-significant Egger's test ($t = -0.62$, $df = 24$, $p = 0.54$).

Study ID	Clinical relevance defined	Input data described	Outcome clearly defined	Reference standard described	Data separation described	Sources of bias identified	Evaluation metrics appropriate	Model explainability addressed	Clinical deployment considered	Reproduc-ibility addressed
Amin 2023	Yes	Yes	Yes	Yes	NA	Yes	Yes	No	Yes	No
Bai 2025	Yes	Yes	Yes	Yes	NA	Yes	Yes	No	Yes	No
Berigan 2024	Yes	Yes	Yes	Yes	NA	Yes	Yes	No	Yes	No
Berzolla 2025	Yes	Yes	Yes	Yes	NA	Yes	Yes	No	Yes	No
Borza 2025	Yes	Yes	Yes	Yes	NA	No	Yes	No	No	No
Butler 2024a	Yes	Yes	Yes	Yes	NA	No	Yes	No	Yes	No
Butler 2024b	Yes	Yes	Yes	Yes	NA	No	Yes	No	Yes	No
Butler 2024c	Yes	Yes	Yes	Yes	NA	No	Yes	No	Yes	No
Çamur 2024	Yes	Yes	Yes	Yes	NA	No	Yes	No	Yes	No
Can 2025	Yes	Yes	Yes	Yes	NA	No	Yes	No	Yes	No
Cesur 2024	Yes	Yes	Yes	Yes	NA	No	Yes	No	Yes	No
Chung 2023	Yes	Yes	Yes	Yes	NA	No	Yes	No	Yes	No
Doshi 2024	Yes	Yes	Yes	Yes	NA	Yes	Yes	No	Yes	No
Güneş 2024	Yes	Yes	Yes	Yes	NA	No	Yes	No	Yes	No
Gupta 2025	Yes	Yes	Yes	Yes	NA	Yes	Yes	No	Yes	No
Jeblick 2022	Yes	Yes	Yes	Yes	NA	No	Yes	No	Yes	No
Kuckelman 2024	Yes	Yes	Yes	Yes	NA	No	Yes	No	Yes	No
Li 2023	Yes	Yes	Yes	Yes	NA	No	Yes	No	Yes	No
Li 2025	Yes	Yes	Yes	Yes	NA	No	Yes	No	Yes	No
Lyu 2023	Yes	Yes	Yes	Yes	NA	No	Yes	No	Yes	No
Maroncelli 2024	Yes	Yes	Yes	Yes	NA	Yes	Yes	No	Yes	No
Park 2024	Yes	Yes	Yes	Yes	NA	No	Yes	No	Yes	No
Pisarcik 2025	Yes	Yes	Yes	Yes	NA	Yes	Yes	No	Yes	No
Prucker 2025	Yes	Yes	Yes	Yes	NA	Yes	Yes	No	Yes	No
Rogasch 2023	Yes	Yes	Yes	Yes	NA	No	Yes	No	Yes	No
Salam 2024	Yes	Yes	Yes	Yes	NA	Yes	Yes	No	Yes	No
Sarangi 2023	Yes	Yes	Yes	Yes	NA	No	Yes	No	Yes	No
Schmidt 2024	Yes	Yes	Yes	Yes	NA	Yes	Yes	No	Yes	No
Stephan 2025	Yes	Yes	Yes	Yes	NA	Yes	Yes	No	Yes	No
Sterling 2024	Yes	Yes	Yes	Yes	NA	No	Yes	No	Yes	No
Sudarshan 2024	Yes	Yes	Yes	Yes	NA	No	Yes	Yes	No	No
Sunshine 2025	Yes	Yes	Yes	Yes	NA	No	Yes	No	Yes	No
Tang 2024	Yes	Yes	Yes	Yes	NA	No	Yes	No	Yes	No

Tariq 2025	Yes	Yes	Yes	Yes	Yes	Yes	Yes	No	Yes	No
Tepe 2024	Yes	Yes	Yes	Yes	NA	No	Yes	No	Yes	No

Supplementary Table 4: MAIC-10 quality assessment

Subgroup analyses

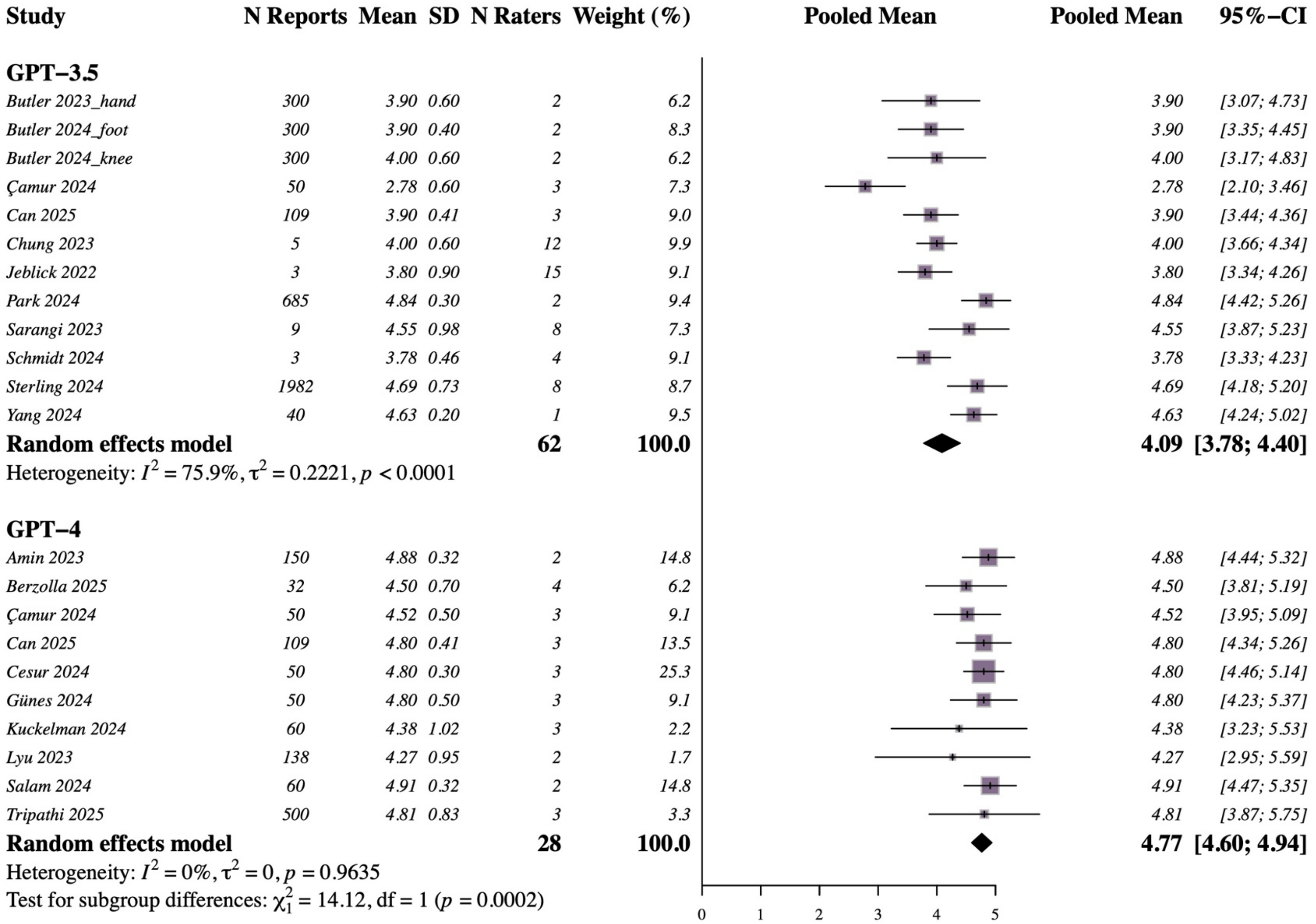

Supplementary Figure 3: Forest plot of medical professionals' accuracy ratings stratified by OpenAI GPT-3.5 vs GPT-4. GPT-4 demonstrated significantly higher accuracy ratings than GPT-3.5 (P < 0.005).

Study	N Reports	Mean	SD	N Raters	Weight (%)	Pooled Mean	95%−CI
**Radiologists**							
Amin 2023	*150*	*4.88*	*0.32*	*2*	*6.6*	*4.88*	*[4.44; 5.32]*
Çamur 2024	*50*	*4.52*	*0.50*	*3*	*5.6*	*4.52*	*[3.95; 5.09]*
Can 2025	*109*	*4.80*	*0.41*	*3*	*6.5*	*4.80*	*[4.34; 5.26]*
Cesur 2024	*50*	*4.80*	*0.30*	*3*	*7.5*	*4.80*	*[4.46; 5.14]*
Günes 2024	*50*	*4.80*	*0.50*	*3*	*5.6*	*4.80*	*[4.23; 5.37]*
Gupta 2025	*50*	*4.56*	*0.50*	*2*	*4.7*	*4.56*	*[3.87; 5.25]*
Jeblick 2022	*3*	*3.80*	*0.90*	*15*	*6.5*	*3.80*	*[3.34; 4.26]*
Kuckelman 2024	*60*	*4.38*	*1.02*	*3*	*2.5*	*4.38*	*[3.23; 5.53]*
Li 2025	*200*	*4.73*	*0.60*	*8*	*6.9*	*4.73*	*[4.31; 5.15]*
Lyu 2023	*138*	*4.27*	*0.95*	*2*	*2.1*	*4.27*	*[2.95; 5.59]*
Maroncelli 2024	*21*	*4.17*	*0.89*	*5*	*4.2*	*4.17*	*[3.39; 4.95]*
Park 2024	*685*	*4.84*	*0.30*	*2*	*6.9*	*4.84*	*[4.42; 5.26]*
Salam 2024	*60*	*4.91*	*0.32*	*2*	*6.6*	*4.91*	*[4.47; 5.35]*
Sarangi 2023	*9*	*4.55*	*0.98*	*8*	*4.8*	*4.55*	*[3.87; 5.23]*
Tang 2024	*100*	*3.74*	*0.04*	*1*	*9.2*	*3.74*	*[3.66; 3.82]*
Tariq 2025	*23*	*4.18*	*0.39*	*3*	*6.7*	*4.18*	*[3.74; 4.62]*
Yang 2024	*40*	*4.63*	*0.20*	*1*	*7.1*	*4.63*	*[4.24; 5.02]*
**Random effects model**				**66**	**100.0**	**4.51**	**[4.29; 4.72]**
Heterogeneity: $I^2 = 90.1\%, \tau^2 = 0.1275, p < 0.0001$							
**Non−Radiologist**							
Bai 2025	*6174*	*4.74*	*0.82*	*2*	*5.9*	*4.74*	*[3.60; 5.88]*
Berzolla 2025	*32*	*4.50*	*0.70*	*4*	*10.4*	*4.50*	*[3.81; 5.19]*
Butler 2023_hand	*300*	*3.90*	*0.60*	*2*	*8.6*	*3.90*	*[3.07; 4.73]*
Butler 2024_foot	*300*	*3.90*	*0.40*	*2*	*12.2*	*3.90*	*[3.35; 4.45]*
Butler 2024_knee	*300*	*4.00*	*0.60*	*2*	*8.6*	*4.00*	*[3.17; 4.83]*
Chung 2023	*5*	*4.00*	*0.60*	*12*	*15.5*	*4.00*	*[3.66; 4.34]*
Rogasch 2023	*6*	*5.00*	*0.05*	*3*	*18.3*	*5.00*	*[4.94; 5.06]*
Sterling 2024	*1982*	*4.69*	*0.73*	*8*	*13.0*	*4.69*	*[4.18; 5.20]*
Tripathi 2025	*500*	*4.81*	*0.83*	*3*	*7.5*	*4.81*	*[3.87; 5.75]*
**Random effects model**				**38**	**100.0**	**4.41**	**[4.07; 4.74]**
Heterogeneity: $I^2 = 86.9\%, \tau^2 = 0.1579, p < 0.0001$							
Test for subgroup differences: $\chi^2_1 = 0.24$, df = 1 ($p = 0.6226$)							

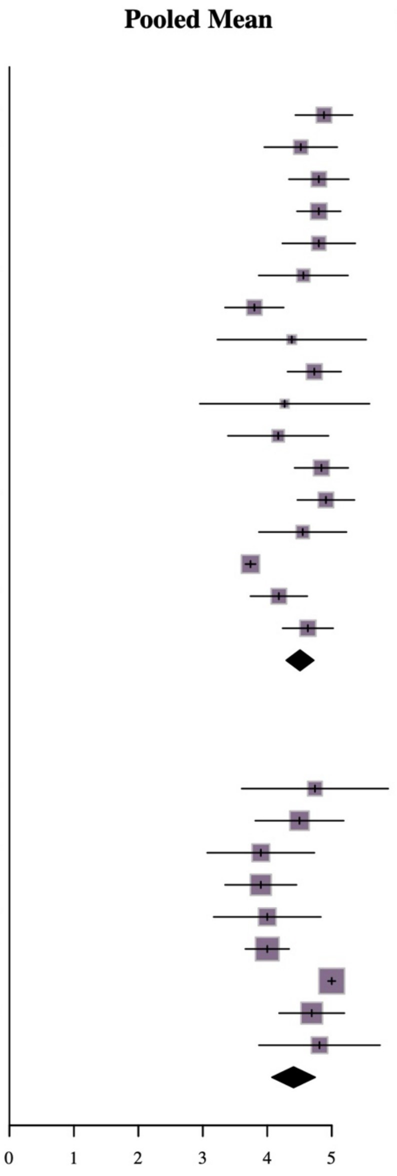

Supplementary Figure 4: Forest plot of medical professionals' accuracy ratings - radiologists vs non-radiologists. Accuracy ratings were similar across assessor groups (P = 0.62).

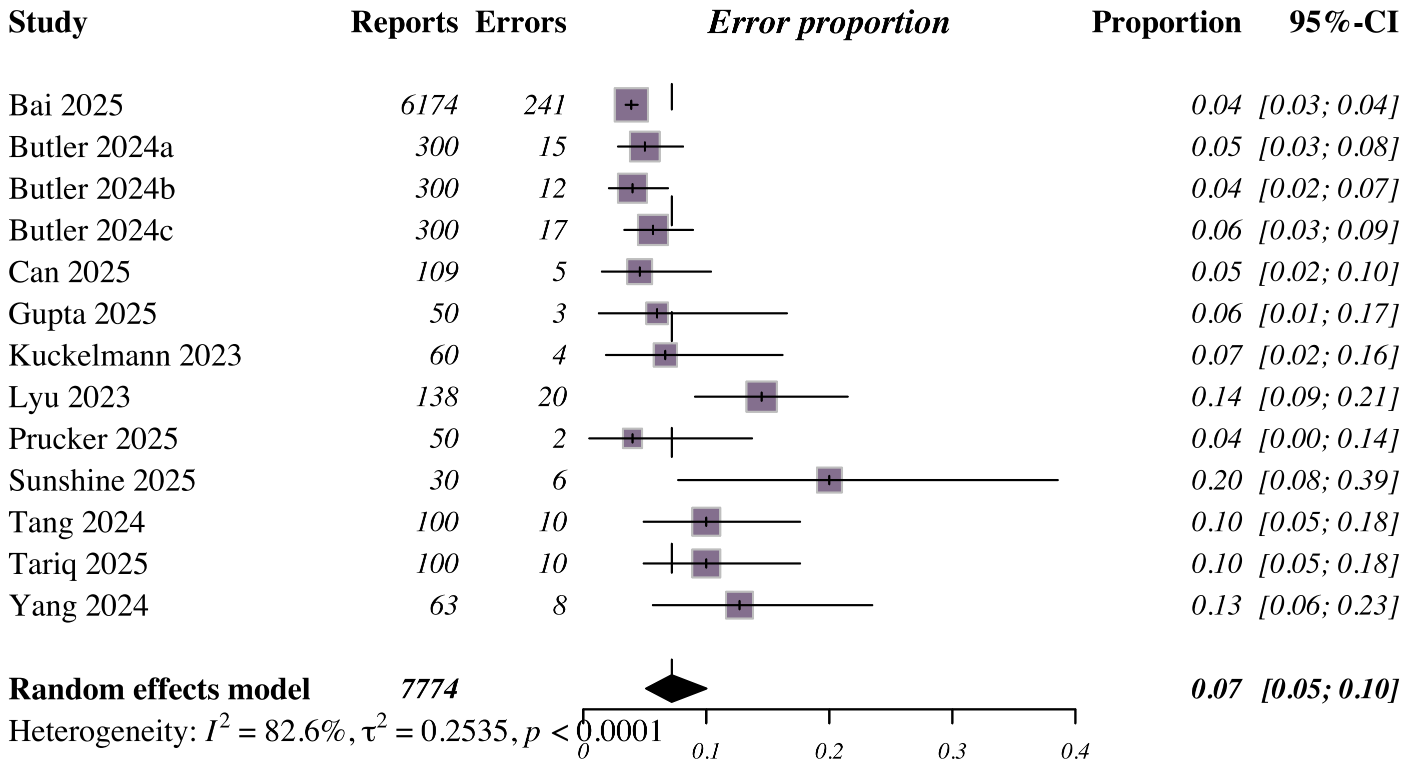

Supplementary Figure 5: Forest plot of error rates in LLM-rewritten reports.

Study	Simplified Mean	Simplified SD	Simplified N	Original Mean	Original SD	Original N	Weight (%)	MD	95%-CI
**XR**									
Butler 2024_foot	8.5	0.4	100	12.2	1.1	100	20.3	-3.70	[-3.93; -3.47]
Butler 2024_hand	7.4	0.7	100	12.6	1.1	100	20.2	-5.20	[-5.46; -4.94]
Butler 2024_knee	7.2	0.6	100	12.7	1.0	100	20.3	-5.50	[-5.73; -5.27]
Doshi 2024	7.6	1.7	150	14.1	2.6	150	19.4	-6.50	[-7.00; -6.00]
Li 2023	5.7	1.1	100	10.2	1.8	100	19.7	-4.50	[-4.91; -4.09]
**Random effects model**			**550**			**550**	**100.0**	**-5.07**	**[-5.99; -4.15]**
Heterogeneity: $I^2 = 97.8\%, \tau^2 = 1.0658, p < 0.0001$									
**CT**									
Butler 2024_foot	8.4	0.6	100	15.4	2.0	100	15.0	-7.00	[-7.41; -6.59]
Butler 2024_hand	7.8	0.7	100	13.4	1.1	100	15.5	-5.60	[-5.86; -5.34]
Butler 2024_knee	7.5	0.5	100	13.4	1.0	100	15.5	-5.90	[-6.12; -5.68]
Doshi 2024	6.8	2.0	150	13.9	2.9	150	14.4	-7.10	[-7.66; -6.54]
Gupta 2025	7.3	3.0	50	12.0	4.0	50	9.9	-4.67	[-6.06; -3.28]
Li 2023	6.0	0.9	100	11.3	1.4	100	15.3	-5.30	[-5.63; -4.97]
Tang 2024	6.6	2.0	100	14.1	2.0	100	14.4	-7.42	[-7.97; -6.87]
**Random effects model**			**700**			**700**	**100.0**	**-6.20**	**[-6.91; -5.48]**
Heterogeneity: $I^2 = 93.6\%, \tau^2 = 0.8524, p < 0.0001$									
**MR**									
Bai 2025	9.6	1.1	6174	11.8	0.7	6174	14.6	-2.20	[-2.23; -2.17]
Butler 2024_foot	8.5	0.5	100	14.1	1.6	100	14.4	-5.60	[-5.93; -5.27]
Butler 2024_hand	7.8	0.7	100	13.8	1.3	100	14.5	-6.00	[-6.29; -5.71]
Butler 2024_knee	7.5	0.6	100	13.5	0.9	100	14.5	-6.00	[-6.21; -5.79]
Chung 2023	4.9	0.7	5	9.6	0.6	5	13.4	-4.70	[-5.51; -3.89]
Doshi 2024	8.2	1.7	150	13.7	2.1	150	14.2	-5.50	[-5.94; -5.06]
Li 2023	6.1	0.9	100	11.1	1.4	100	14.4	-5.00	[-5.33; -4.67]
**Random effects model**			**6729**			**6729**	**100.0**	**-5.00**	**[-6.00; -4.00]**
Heterogeneity: $I^2 = 99.8\%, \tau^2 = 1.7855, p = 0$									
**Multi-modality**									
Li 2025	7.0	1.2	200	10.7	1.0	200	26.9	-3.70	[-3.92; -3.48]
Sudarshan 2024	3.6	2.0	16	11.0	2.0	16	22.5	-7.38	[-8.77; -5.99]
Sunshine 2025	7.2	1.1	30	12.5	1.6	30	25.6	-5.30	[-6.01; -4.59]
Yang 2024	8.5	2.0	40	12.3	2.0	40	25.0	-3.80	[-4.67; -2.92]
**Random effects model**			**286**			**286**	**100.0**	**-4.96**	**[-6.57; -3.36]**
Heterogeneity: $I^2 = 92.9\%, \tau^2 = 2.4872, p < 0.0001$									

Test for subgroup differences: $\chi^2_3 = 5.83$, df = 3 ($p = 0.1200$)

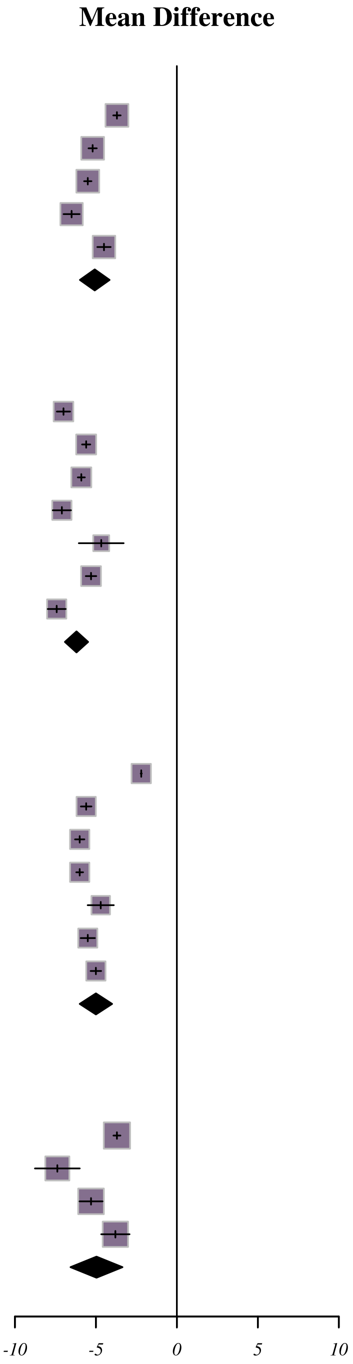

Supplementary Figure 6: Forest plot of pooled mean differences in Flesch–Kincaid Grade Level (FKGL) scores.

Forest plot comparing simplified versus original radiology reports, stratified by imaging modality. Negative values indicate lower FKGL scores, reflecting improved readability for simplified reports.

		Original Reports				Simplified Reports			
**Modality**	**Metric**	**Mean**	**95% CI**	**Studies**	**Reports**	**Mean**	**95% CI**	**Studies**	**Reports**
**CT**	ARI	20.0	19.4–20.6	1	107	9.1	6.3–12.0	2	157
	FKGL	13.4	12.4–14.4	7	700	7.3	6.7–7.9	8	750
	FRES	28.0	25.5–30.5	4	400	73.6	66.7–80.4	4	400
**MRI**	ARI	13.5	6.8–20.1	2	107	6.1	3.9–8.2	2	107
	FKGL	12.5	11.3–13.8	7	6,729	7.5	6.4–8.7	7	6,729
	FRES	26.3	21.2–31.4	5	6,574	67.7	55.9–79.6	5	6,574
**Multi-modality**	ARI	9.9	9.3–10.6	1	40	7.8	4.2–11.5	2	140
	FKGL	11.6	10.7–12.6	4	286	7.7	5.8–9.6	6	425
	FRES	31.8	11.8–51.8	2	230	66.1	60.4–71.7	5	469
**XR**	ARI	13.3	13.2–13.5	2	545	8.3	3.5–13.0	2	545
	FKGL	12.4	11.1–13.6	5	550	7.3	6.4–8.2	5	550
	FRES	37.1	35.1–39.1	5	900	77.8	73.7–82.0	5	900

Supplementary Table 5: Meta-analytic pooled readability scores for original and LLM-rewritten radiology reports.
Readability scores (mean ± 95% CI) are presented by modality (XR vs. CT/MR) and scoring system (Flesch Kincaid Grade Level (FKGL), Flesch Reading Ease (FRES), Automated Readability Index (ARI)). Values include number of contributing studies and total number of reports per group.

Report lengths

The pooled mean word counts for original radiology reports were 49.8 (SD = 23.8; 4 studies; 400 reports) for X-ray, 192.0 (SD = 44.9; 6 studies; 463 reports) for CT, and 198.0 (SD = 61.6; 9 studies; 543 reports) for MR. In comparison, the corresponding mean word counts for simplified reports were 155.0 (SD = 48.2; 4 studies; 400 reports) for X-ray, 273.0 (SD = 38.3; 6 studies; 463 reports) for CT, and 262.0 (SD = 65.8; 9 studies; 543 reports) for MR.
This represents an increase in report length of 211% for X-ray, 42% for CT, and 32% for MR from the original report to the LLM-simplified report. A graphical comparison of report lengths across modalities and exam types is provided in **Supplementary Figure 5.**

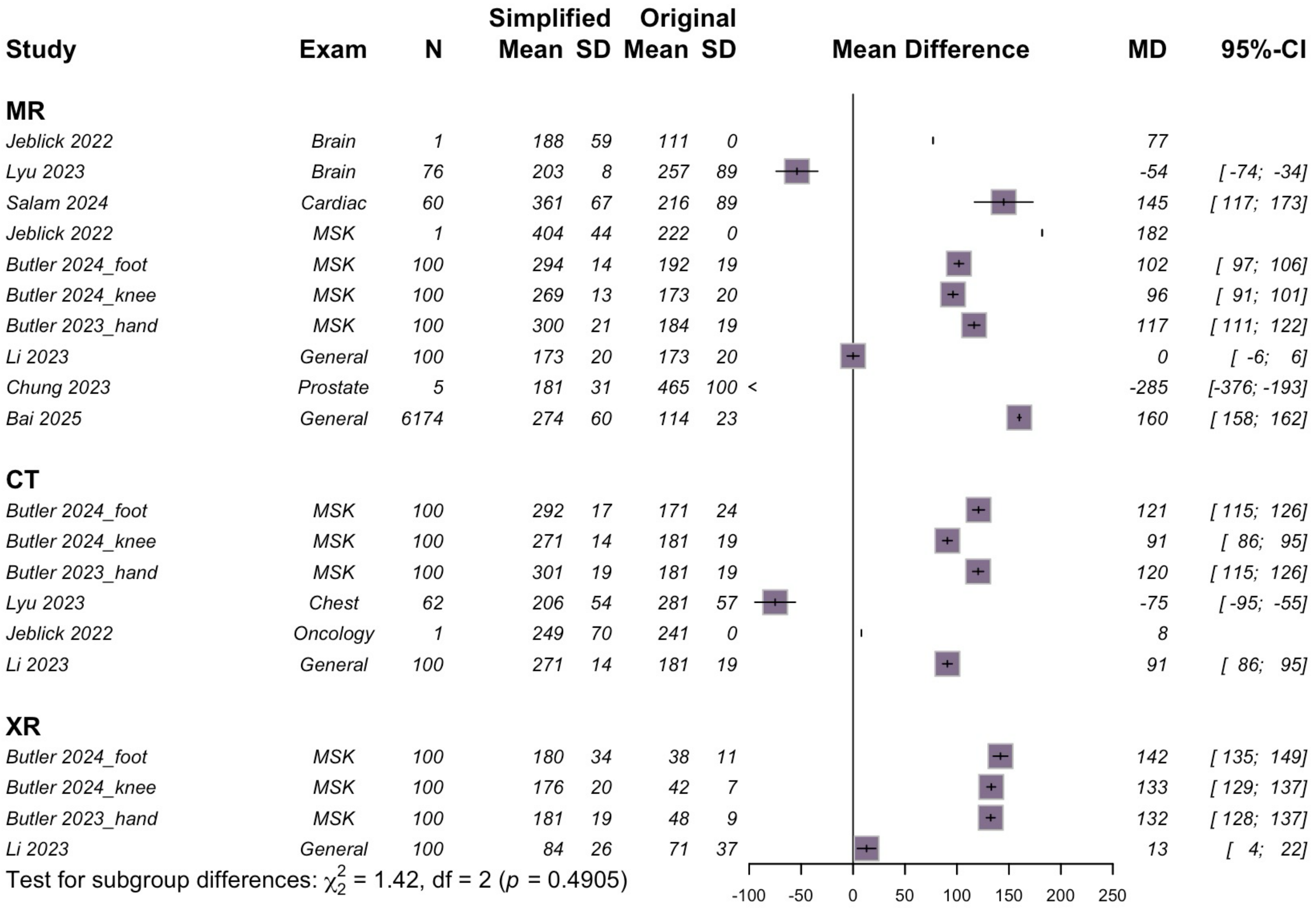

Supplementary Figure 7: Forest plot showing word counts for original and LLM-rewritten reports.
Forest plot stratified by imaging modality and examination type. Each point represents the mean word count per study.

LLM comparisons

13 studies compared the performance of different LLMs to GPT-4 **(Supplementary Table 5)**. A pooled analysis using robust variance estimation across multiple outcome measures (including accuracy, simplicity, completeness, understandability, and empathy) demonstrated that GPT-4 consistently outperformed other LLMs in both medical professional and patient assessments. Claude-3-Opus performed closest to GPT-4 across all outcomes with a MD in Likert score of –0.32 (95% CI: –0.66 to –0.03) followed by Gemini-1.5 Ultra MD of –0.58 (95% CI: –0.85 to –0.31). Llama-3-70B, was comparable to GPT-4 for simplification but worse for empathy, with an overall MD of –1.29 (95% CI: –1.94 to -0.64). The comparisons for individual outcomes are presented in **Supplementary Figure 6 (page 12)**.

DeepSeek-R1 (DeepSeek, Hangzhou, Zhejiang Province, China) was compared to the free version of OpenAI GPT o1-preview in 6,174 oncology MRI reports and showed a better accuracy rating by two oncologists 4.84 ± 0.57 vs 4.74 ± 0.82 (P < 0.001) and small error rate 0.6% vs 3.9% [33].

One study fine-tuned a T5 (770M) model on 4,000 chest CT reports paired with LLaMA 2 13B–generated lay summaries that were manually checked for errors [65]. Neither the fine-tuned model nor the dataset is publicly available. Compared with LLaMA 2, the fine-tuned model showed lower rates of hallucination (6% vs 18%) and missing information (4% vs 17%), as assessed by radiologists. In lay user testing, comprehension of reports improved by 63% relative to the original reports.

FKGL readability scores did not differ significantly for Claude-3-Opus (MD = 0.52; 95% CI: –1.72 to 2.76; 3 studies; 209 reports) and Gemini (MD = 0.01; 95% CI: –1.70 to 1.71; 3 studies; 209 reports) compared with GPT-4. Scores were significantly better for Llama-3-70B (MD = –0.60; 95% CI: –1.07 to –0.13; 2 studies; 100 reports) and worse for Mixtral-8x7B (MD = 1.16; 95% CI: 0.91 to 1.40; 2 studies; 159 reports) and Mistral-7B (MD = 1.17; 95% CI: –0.10 to 2.45; 2 studies; 159 reports) (**Supplementary Figure 7, page 13**).

Model	Mean difference from GPT-4 (95% CI)	Studies (n)	Reports (N)	Raters (N)
**Claude-3-Opus**	-0.32 [-0.66; -0.03]	5	309	15
**Gemini-1.5 Ultra**	-0.58 [-0.85; -0.31]	6	336	55
**Mistral-7B**	-0.63 [-0.66; -0.61]	2	159	6
**Mixtral-8x7B**	-1.33 [-2.27; -0.40]	2	159	6
**Llama-3-70b**	-1.29 [-1.94; -0.64]	1	50	3
**Google BARD**	-0.05 [-0.12; 0.02]	1	750	2

Supplementary Table 6: Comparison of various LLMs with GPT-4 across multiple outcomes.
Outcomes assessed by clinicians and patients (accuracy, completeness, simplicity, empathy). Note: Negative mean differences favour GPT-4; positive values favour the comparator LLM.

Study	Outcome	N	LLM Mean	LLM SD	GPT−4 Mean	GPT−4 SD	MD	95%−CI
**Gemini−Ultra**								
Can 2025	*Accuracy*	*109*	*4.01*	*0.56*	*4.80*	*0.41*	*−0.79*	*[−0.92; −0.66]*
Çamur 2024	*Accuracy*	*50*	*4.32*	*0.60*	*4.52*	*0.50*	*−0.20*	*[−0.42; 0.02]*
Günes 2024	*Accuracy*	*50*	*4.80*	*0.50*	*4.80*	*0.50*	*0.00*	*[−0.20; 0.20]*
Cesur 2024	*Accuracy*	*50*	*4.90*	*0.10*	*4.80*	*0.30*	*0.10*	*[0.01; 0.19]*
Prucker 2025	*Simplicity*	*50*	*2.82*	*0.77*	*3.69*	*0.59*	*−0.87*	*[−1.14; −0.60]*
Can 2025	*Simplicity*	*109*	*3.87*	*0.54*	*4.70*	*0.50*	*−0.83*	*[−0.97; −0.69]*
Can 2025	*Completeness*	*109*	*3.98*	*0.47*	*4.74*	*0.48*	*−0.76*	*[−0.89; −0.63]*
Pisarcik 2025	*Understandability*	*27*	*3.68*	*0.92*	*4.28*	*0.77*	*−0.60*	*[−1.05; −0.15]*
Pisarcik 2025	*Empathy*	*27*	*3.10*	*0.97*	*3.87*	*0.71*	*−0.77*	*[−1.22; −0.32]*
Prucker 2025	*Empathy*	*50*	*2.65*	*0.74*	*3.85*	*0.89*	*−1.20*	*[−1.52; −0.88]*
**Claude−3−Opus**								
Can 2025	*Accuracy*	*109*	*4.72*	*0.51*	*4.80*	*0.41*	*−0.08*	*[−0.20; 0.04]*
Çamur 2024	*Accuracy*	*50*	*4.70*	*0.40*	*4.52*	*0.50*	*0.18*	*[0.00; 0.36]*
Günes 2024	*Accuracy*	*50*	*4.70*	*0.60*	*4.80*	*0.50*	*−0.10*	*[−0.32; 0.12]*
Cesur 2024	*Accuracy*	*50*	*4.80*	*0.50*	*4.80*	*0.30*	*0.00*	*[−0.16; 0.16]*
Prucker 2025	*Simplicity*	*50*	*3.15*	*0.57*	*3.69*	*0.59*	*−0.54*	*[−0.77; −0.31]*
Can 2025	*Simplicity*	*109*	*4.77*	*0.47*	*4.70*	*0.50*	*0.07*	*[−0.06; 0.20]*
Can 2025	*Completeness*	*109*	*3.79*	*0.41*	*4.74*	*0.48*	*−0.95*	*[−1.07; −0.83]*
Prucker 2025	*Empathy*	*50*	*2.67*	*0.84*	*3.85*	*0.89*	*−1.18*	*[−1.52; −0.84]*
**Mistral−7B**								
Prucker 2025	*Simplicity*	*50*	*2.24*	*0.71*	*3.69*	*0.59*	*−1.45*	*[−1.71; −1.19]*
Can 2025	*Simplicity*	*109*	*3.79*	*0.45*	*4.70*	*0.50*	*−0.91*	*[−1.04; −0.78]*
Can 2025	*Completeness*	*109*	*3.91*	*0.35*	*4.74*	*0.48*	*−0.83*	*[−0.94; −0.72]*
Prucker 2025	*Empathy*	*50*	*1.64*	*0.79*	*3.85*	*0.89*	*−2.21*	*[−2.54; −1.88]*
**Mixtral−8x7B**								
Prucker 2025	*Simplicity*	*50*	*2.21*	*0.53*	*3.69*	*0.59*	*−1.48*	*[−1.70; −1.26]*
Can 2025	*Simplicity*	*109*	*3.76*	*0.47*	*4.70*	*0.50*	*−0.94*	*[−1.07; −0.81]*
Can 2025	*Completeness*	*109*	*3.76*	*0.43*	*4.74*	*0.48*	*−0.98*	*[−1.10; −0.86]*
Prucker 2025	*Empathy*	*50*	*2.08*	*0.27*	*3.85*	*0.89*	*−1.77*	*[−2.03; −1.51]*
**Llama−3−70b**								
Prucker 2025	*Simplicity*	*50*	*3.72*	*0.65*	*3.69*	*0.59*	*0.03*	*[−0.21; 0.27]*
Prucker 2025	*Empathy*	*50*	*2.53*	*0.75*	*3.85*	*0.89*	*−1.32*	*[−1.64; −1.00]*
**BARD**								
Amin 2023	*Accuracy*	*750*	*4.79*	*0.44*	*4.88*	*0.32*	*−0.09*	*[−0.13; −0.05]*
Amin 2023	*Releasability*	*750*	*4.47*	*0.13*	*4.49*	*0.09*	*−0.02*	*[−0.03; −0.01]*

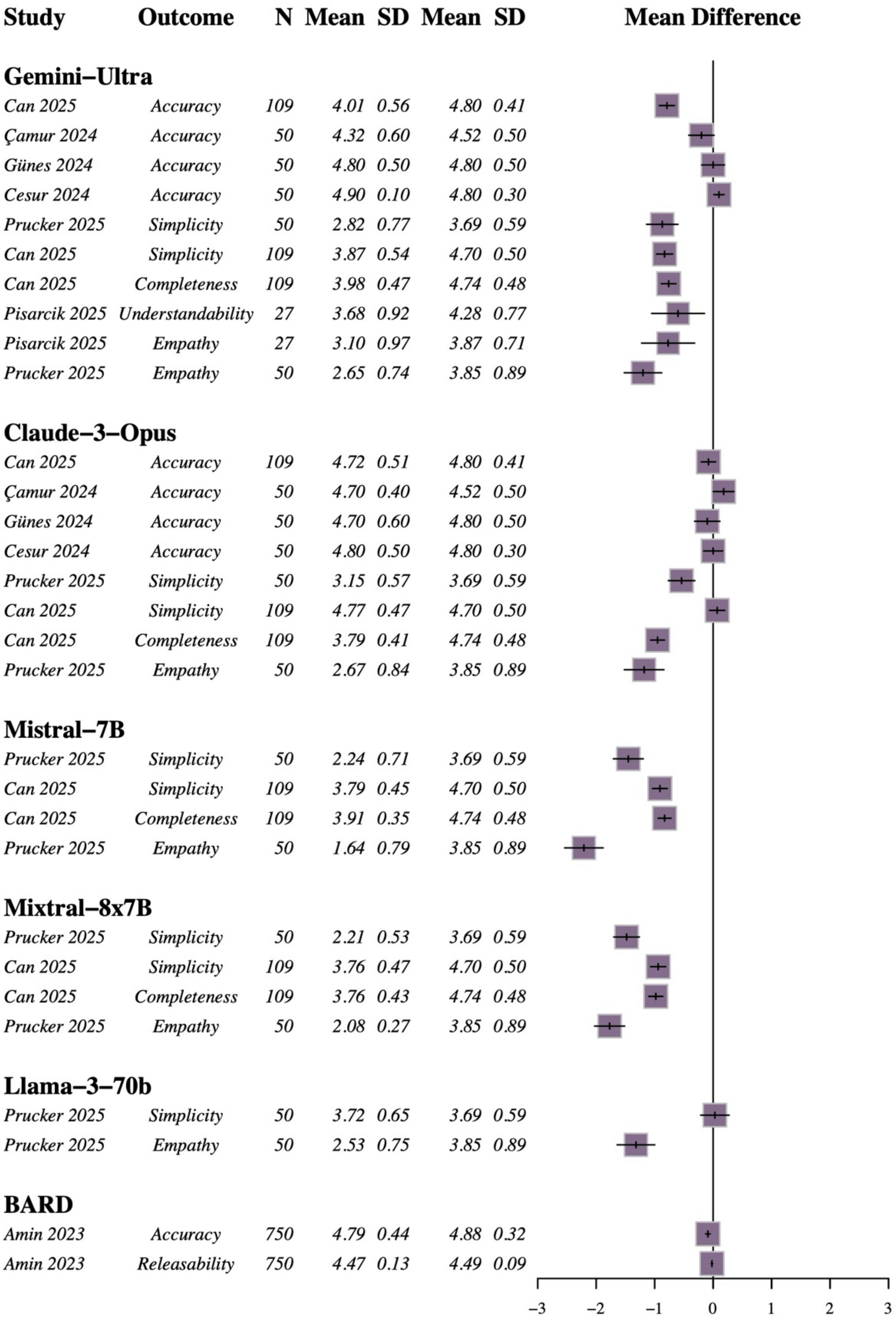

Supplementary Figure 8: Forest plot comparing the performance of LLMs with GPT-4 across multiple outcomes. Negative values favour GPT-4, positive values favour the comparator LLM.

Study	Outcome	N	LLM Mean	LLM SD	GPT−4 Mean	GPT−4 SD	MD	95%−CI
**Gemini−Ultra**								
Can 2025	*Readability*	*109*	*11.60*	*1.63*	*10.70*	*0.96*	*0.90*	*[0.54; 1.26]*
Gupta 2025	*Readability*	*50*	*5.51*	*1.90*	*7.35*	*3.00*	*−1.84*	*[−2.82; −0.86]*
Prucker 2025	*Readability*	*50*	*8.70*	*1.11*	*7.90*	*1.33*	*0.80*	*[0.32; 1.28]*
**Random effects model**		**209**					**0.01**	**[−1.70; 1.71]**
**Claude−3−Opus**								
Can 2025	*Readability*	*109*	*12.20*	*0.90*	*10.70*	*0.96*	*1.50*	*[1.25; 1.75]*
Gupta 2025	*Readability*	*50*	*5.53*	*1.30*	*7.35*	*3.00*	*−1.82*	*[−2.73; −0.91]*
Prucker 2025	*Readability*	*50*	*9.70*	*1.56*	*7.90*	*1.33*	*1.80*	*[1.23; 2.37]*
**Random effects model**		**209**					**0.52**	**[−1.72; 2.76]**
**Mixtral−8x7B**								
Can 2025	*Readability*	*109*	*11.90*	*0.96*	*10.70*	*0.96*	*1.20*	*[0.95; 1.45]*
Prucker 2025	*Readability*	*50*	*8.70*	*2.30*	*7.90*	*1.33*	*0.80*	*[0.06; 1.54]*
**Random effects model**		**159**					**1.16**	**[0.91; 1.40]**
**Llama−3−70b**								
Gupta 2025	*Readability*	*50*	*6.43*	*1.70*	*7.35*	*3.00*	*−0.92*	*[−1.88; 0.04]*
Prucker 2025	*Readability*	*50*	*7.40*	*1.41*	*7.90*	*1.33*	*−0.50*	*[−1.04; 0.04]*
**Random effects model**		**100**					**−0.60**	**[−1.07; −0.13]**
**Mistral−7B**								
Can 2025	*Readability*	*109*	*12.50*	*1.04*	*10.70*	*0.96*	*1.80*	*[1.53; 2.07]*
Prucker 2025	*Readability*	*50*	*8.40*	*1.41*	*7.90*	*1.33*	*0.50*	*[−0.04; 1.04]*
**Random effects model**		**159**					**1.17**	**[−0.10; 2.45]**

Test for subgroup differences: $\chi^2_4 = 43.47$, df = 4 ($p < 0.0001$)

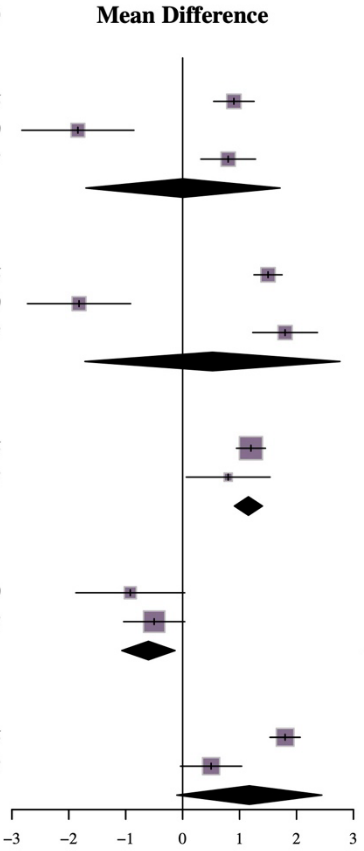

Supplementary Figure 9: Forest plot comparing LLMs with GPT-4 for FKGL readability scores.
Positive values indicate more difficult readability and favour GPT-4, while negative values indicate easier readability scores and favour the comparator LLM.

Prompting strategies

Of the 38 studies, nearly all (36/38, 95%) explicitly prompted the LLM to simplify the report text. Context (e.g., specifying the audience such as "patient" or "layperson") was included in 30/38 studies (79%). Output constraints were present in 31/38 studies (82%); within this group. 10/31 (31%) specified a target reading level or age, and 7/31 (23%) included explicit safety instructions (such as no omissions, no speculation, avoid assumptions). A role was assigned to the LLM in 2/38 studies (5%) (e.g. helpful Radiology Report Simplifier or expert at explaining medical information), while 3/38 studies (8%) used few-shot examples of simplified reports. Two studies (5%) did not report their prompt verbatim. Prompt details and assessment are provided in **Supplementary Table 6.**

Ten studies reported experimenting with several prompts prior to their finalised prompt [43–45,47,48,54,59,60,64,65]. However, only three studies compared performance of LLMs with different prompts[44,45,60]. Both Gupta et al. 2025 and Doshi et al. 2025 found that more detailed prompting resulted in improvement of readability scores. For example adding "simplify at 7th-grade level" to the GPT-4 prompt reduced the average reading level from 9.3 (7.6 - 10.9) to 7.5 (6.1–8.9) ([44,45,60]), adding "explain it to a 15-year-old" reduced the FKGL from 10.49 to 7.35 ([44,45,60]) and adding "make it understandable for all educational backgrounds" improved the mean FRE score from 55 to 56.5 ($P < .05$) ([44,45,60]). However, Stephan et al. 2025 did not find a significant difference in patient assessment by adding "make it understandable for all educational backgrounds" to the prompt ([44,45,60]). In Tang et al. 2025, adding medical knowledge to the prompt such as relevant entities and their definitions reduced incorrect outputs from 36% to 10% [64].

Study	Prompt	Context	Role	Action	Output	Example
Amin 2023 [1]	Simplify this radiology report.	—	—	✓	—	—
Bai 2025 [2]	Interpret the reports in a manner understandable to patients without a medical background	✓	—	✓	✓	—
Berigan 2024 [3]	Explain this radiology report to a patient in layman's terms in second person.	✓	—	✓	✓	—
Berzolla 2025 [4]	I don't understand what this MRI report is saying. Can you please simplify and explain the contents?	✓	—	✓	—	—
Borza 2025 [5]	Can you explain my radiology report?	—	—	✓	—	—
Butler 2024a[6],b[7],c[8]	Explain this radiology report to a patient in layman's terms in the second person.	✓	—	✓	✓	—
Çamur 2024 [9]	I will write the findings from the CT report below. Please explain them in a way that someone without a medical background can understand	✓	—	✓	✓	—
Can 2025 [10]	Transform technical findings into simplified, patient-friendly summaries; no omissions/no speculation; reassuring tone; bracket lay definitions when needed; Use template with Findings, Impression, Recommendations.	✓	—	✓	✓	—
Cesur 2024 [11]	I will write the MRI findings below. Please explain them so someone without medical background can understand.	✓	—	✓	✓	—
Chung 2023 [12]	Summarize the radiology report in letter format to a patient at a 6th-grade reading level. Include size/location of lesion and likelihood of malignancy if applicable.	✓	—	✓	✓	—
Doshi 2024 [13]	P1: Simplify this radiology report.	—	—	✓	—	—
	P2: Simplify this radiology report. I am a patient.	✓	—	✓	—	—
	P3: Simplify this radiology report at the 7th-grade level.	—	—	✓	✓	—
Güneş 2024 [14]	I will write the ultrasound findings below. Please explain them so someone without medical background can understand.	✓	—	✓	✓	—
Gupta 2025 [15]	P1: You are a helpful Radiology Report Simplifier. Simplify this radiology report. In your response, do not give any extra text and do not add any paragraphs. Only give the simplified report in the response.	—	✓	✓	✓	—
	P2: P1 + Simplify this radiology report to layman language.	✓	✓	✓	✓	—
	P3: P1 + Simplify this radiology report to explain it to a 15-year-old.	✓	✓	✓	✓	—
	P4: P1 + Your primary role is to simplify CT radiology reports into easy language for explaining to a 15-year-old. To help you with the task, you have been provided with three sample original reports along with their simplified versions. You should try to give your response in line with these examples. Sample reports (x 3)	✓	✓	✓	✓	✓
	P5: P4 + Below the examples, you have also been given a list of medical terms and their desired English simplifications. You should strictly use them as reference for simplification and use only these if you encounter the specified medical terms in the original reports given to you, to ensure accuracy and consistency.	✓	✓	✓	✓	✓
Jeblick 2022 [16]	Explain this medical report to a child using simple language.	✓	—	✓	✓	—
Kuckelman 2024 [17]	Produce an organized and concise layperson summary of the findings of the following radiology report.	✓	—	✓	✓	—
Li 2023 [18]	Explain this radiology report to a patient in layman's terms in second person:	✓	—	✓	✓	—
Li 2025 [19]	Please explain the following procedural/surgical report to a person as if it is the patient's report using simple terms like a child. Write concisely in second person and separate into: Description; Findings; Technically successful; Complications; Follow-up.	✓	—	✓	✓	—
Lyu 2023 [20]	Translate a radiology report into plain language that is easy to understand.	—	—	✓	✓	—

Maroncelli 2024 [21]	Explain this medical report to a patient using simple language.	✓	—	✓	✓	—
Park 2024 [22]	Please make it easy for patients.	✓	—	✓	✓	—
Pisarcik 2025 [23]	Simplify this medical report so that it is clear, correct, and easily understandable by the patient without requiring any medical expertise.	✓	—	✓	✓	—
Prucker 2025 [24]	Transform technical findings into patient-friendly summaries; if a word can't be simplified, add a lay explanation in brackets.	✓	—	✓	✓	—
Rogasch 2023 [25]	Please explain my PET report to me.	✓	—	✓	—	—
Salam 2024 [26]	Explain the radiology report in a language understandable to a medical layperson.	✓	—	✓	✓	—
Sarangi 2023 [27]	Explain this medical report in a simplified language	—	—	✓	✓	—
Schmidt 2024 [28]	Explain the following MRI report of the knee joint in simple language.	—	—	✓	✓	—
Stephan 2025 [29]	P1: Rewrite the radiology report to make it easier for a patient to understand. Do not leave out any information or content.	✓	—	✓	✓	—
	P2: Rewrite the radiology report to make it understandable for patients of all educational backgrounds. Do not leave any information or content.	✓	—	✓	✓	—
Sterling 2024 [30]	Summarize radiology results at a basic (fifth grade) reading level, avoid making assumptions or evaluations beyond what is explicitly stated in the report, translate any medical jargon into simple language, define necessary medical terms, and describe the imaging modality. Several examples of ideal LLM-generated summaries (i.e., few shot prompting) and was concatenated with deidentified radiology reports.	✓	—	✓	✓	✓
Sudarshan 2024 [31]	Described but not reported.	—	—	—	—	—
Sunshine 2025 [32]	Please rewrite the following radiology report as a patient-friendly summary between 100 and 175 words. Start with a simple overall impression statement using natural language that is easy to understand. Focus on concisely summarizing the most relevant findings, conclusions, and recommended next steps. Minimize medical jargon, but clearly define any complex technical terms needed for accuracy. Provide enough details and context so that a layperson understands the key results and implications of the radiology report. Accurately reflect any potential abnormalities rather than omitting them. The summary should strike a balance between brevity and inclusion of significant information from the original report. The goal is an accessible and easy to understand summary that avoids oversimplification and remains clinically aligned with objective findings. Ensure the significance and severity of any abnormalities are accurately represented without downplaying or exaggerating. Only include next steps if you are highly confident, they are appropriate recommendations for the patient.	✓	—	✓	✓	—
Tang 2024 [33]	Please translate this radiology report into a plain language summary that is easy to understand by someone with only a fifth-grade education. Ensure that the generated summary is under four sentences. Do not make anything up about the patient's report. Here is the original report: Here are some examples of original reports and their translations:	✓	—	✓	✓	✓
Tariq 2025 [34]	You are an expert at explaining medical information to people with little medical knowledge. Given the impressions of a radiologist from a radiology exam below, please generate a layman summary of this information. Please replace all medical terminology with simple layman terms. Please make the explanation as brief as possible. Please do not use any non-essential information or pleasantries or any information not explicitly mentioned.	✓	✓	✓	✓	—
Tepe 2024 [35]	Translate the radiology report into plain language that is easy to understand.	✓	—	✓	✓	—
Tripathi 2024 [36]	Generate a paragraph summarizing the report text at a 6th-grade level and in a patient-friendly manner.	✓	—	✓	✓	—
van Driel 2025 [37]	Described but not reported.	—	—	—	—	—
Yang 2024 [38]	List complicated medical terms and provide lay explanations. Finally, write a simplification of the original sentence.	✓	—	✓	✓	—

Supplementary Table 7: LLM prompts used in included studies

PRISMA checklist

Section and Topic	Item #	Checklist item	Location where item is reported
**TITLE**			
Title	1	Identify the report as a systematic review.	Title
**ABSTRACT**			
Abstract	2	See the PRISMA 2020 for Abstracts checklist.	Abstract
**INTRODUCTION**			
Rationale	3	Describe the rationale for the review in the context of existing knowledge.	Introduction
Objectives	4	Provide an explicit statement of the objective(s) or question(s) the review addresses.	Introduction
**METHODS**			
Eligibility criteria	5	Specify the inclusion and exclusion criteria for the review and how studies were grouped for the syntheses.	Methods
Information sources	6	Specify all databases, registers, websites, organisations, reference lists and other sources searched or consulted to identify studies. Specify the date when each source was last searched or consulted.	Methods
Search strategy	7	Present the full search strategies for all databases, registers and websites, including any filters and limits used.	Supplementary Materials
Selection process	8	Specify the methods used to decide whether a study met the inclusion criteria of the review, including how many reviewers screened each record and each report retrieved, whether they worked independently, and if applicable, details of automation tools used in the process.	Methods
Data collection process	9	Specify the methods used to collect data from reports, including how many reviewers collected data from each report, whether they worked independently, any processes for obtaining or confirming data from study investigators, and if applicable, details of automation tools used in the process.	Methods
Data items	10a	List and define all outcomes for which data were sought. Specify whether all results that were compatible with each outcome domain in each study were sought (e.g. for all measures, time points, analyses), and if not, the methods used to decide which results to collect.	Methods
	10b	List and define all other variables for which data were sought (e.g. participant and intervention characteristics, funding sources). Describe any assumptions made about any missing or unclear information.	Methods
Study risk of bias assessment	11	Specify the methods used to assess risk of bias in the included studies, including details of the tool(s) used, how many reviewers assessed each study and whether they worked independently, and if applicable, details of automation tools used in the process.	Methods
Effect measures	12	Specify for each outcome the effect measure(s) (e.g. risk ratio, mean difference) used in the synthesis or presentation of results.	Methods
Synthesis methods	13a	Describe the processes used to decide which studies were eligible for each synthesis (e.g. tabulating the study intervention characteristics and comparing against the planned groups for each synthesis (item #5)).	Methods
	13b	Describe any methods required to prepare the data for presentation or synthesis, such as handling of missing summary statistics, or data conversions.	Methods
	13c	Describe any methods used to tabulate or visually display results of individual studies and syntheses.	Methods
	13d	Describe any methods used to synthesize results and provide a rationale for the choice(s). If meta-analysis was performed, describe the model(s), method(s) to identify the presence and extent of statistical heterogeneity, and software package(s) used.	Methods
	13e	Describe any methods used to explore possible causes of heterogeneity among study results (e.g. subgroup analysis, meta-regression).	NA
	13f	Describe any sensitivity analyses conducted to assess robustness of the synthesized results.	NA

Section and Topic	Item #	Checklist item	Location where item is reported
Reporting bias assessment	14	Describe any methods used to assess risk of bias due to missing results in a synthesis (arising from reporting biases).	NA
Certainty assessment	15	Describe any methods used to assess certainty (or confidence) in the body of evidence for an outcome.	Methods
**RESULTS**			
Study selection	16a	Describe the results of the search and selection process, from the number of records identified in the search to the number of studies included in the review, ideally using a flow diagram.	Results
	16b	Cite studies that might appear to meet the inclusion criteria, but which were excluded, and explain why they were excluded.	Results
Study characteristics	17	Cite each included study and present its characteristics.	Results – characteristics of included studies
Risk of bias in studies	18	Present assessments of risk of bias for each included study.	Results - Quality assessment
Results of individual studies	19	For all outcomes, present, for each study: (a) summary statistics for each group (where appropriate) and (b) an effect estimate and its precision (e.g. confidence/credible interval), ideally using structured tables or plots.	Results
Results of syntheses	20a	For each synthesis, briefly summarise the characteristics and risk of bias among contributing studies.	Results and Supplementary Material
	20b	Present results of all statistical syntheses conducted. If meta-analysis was done, present for each the summary estimate and its precision (e.g. confidence/credible interval) and measures of statistical heterogeneity. If comparing groups, describe the direction of the effect.	Results
	20c	Present results of all investigations of possible causes of heterogeneity among study results.	NA
	20d	Present results of all sensitivity analyses conducted to assess the robustness of the synthesized results.	NA
Reporting biases	21	Present assessments of risk of bias due to missing results (arising from reporting biases) for each synthesis assessed.	NA
Certainty of evidence	22	Present assessments of certainty (or confidence) in the body of evidence for each outcome assessed.	NA
**DISCUSSION**			
Discussion	23a	Provide a general interpretation of the results in the context of other evidence.	Discussion
	23b	Discuss any limitations of the evidence included in the review.	Discussion
	23c	Discuss any limitations of the review processes used.	Discussion
	23d	Discuss implications of the results for practice, policy, and future research.	Discussion
**OTHER INFORMATION**			
Registration and protocol	24a	Provide registration information for the review, including register name and registration number, or state that the review was not registered.	Abstract and Methods
	24b	Indicate where the review protocol can be accessed, or state that a protocol was not prepared.	Abstract and Methods
	24c	Describe and explain any amendments to information provided at registration or in the protocol.	NA
Support	25	Describe sources of financial or non-financial support for the review, and the role of the funders or sponsors in the review.	Abstract
Competing interests	26	Declare any competing interests of review authors.	NA
Availability of data, code and other materials	27	Report which of the following are publicly available and where they can be found: template data collection forms; data extracted from included studies; data used for all analyses; analytic code; any other materials used in the review.	NA

Supplementary Table 9: PRISMA checklist [37]

References of included studies

1 Amin KS, Davis MA, Doshi R, Haims AH, Khosla P, Forman HP. Accuracy of ChatGPT, Google Bard, and Microsoft Bing for simplifying radiology reports. *Radiology* 2023; 309: e232561.

2 Bai X, Feng M, Ma W, Liao Y. Application of artificial intelligence chatbots in interpreting magnetic resonance imaging reports: a comparative study. *Sci Rep* 2025; 15: 31266.

3 Berigan K, Short R, Reisman D, et al. The impact of large language model-generated radiology report summaries on patient comprehension: a randomized controlled trial. *J Am Coll Radiol* 2024; 21: 1898–903.

4 Berzolla E, Gosnell GG, Chen L, Vonck C, Alaia E, Meislin R. Artificial intelligence large language models improve patient comprehension of radiologist magnetic resonance imaging reports. *Arthroscopy* 2025; 41: 4607–14.

5 Bozer A, Pekçevik Y. Comparative evaluation of large language models in explaining radiology reports: expert assessment of readability, understandability, and communication features. *Insights Imaging* 2025; 16: 232.

6 Butler JJ, Acosta E, Kuna MC, et al. Decoding radiology reports: artificial intelligence–large language models can improve the readability of hand and wrist orthopedic radiology reports. *Hand* 2025; 20: 1144–52.

7 Butler JJ, Puleo J, Harrington MC, et al. From technical to understandable: artificial intelligence large language models improve the readability of knee radiology reports. *Knee Surg Sports Traumatol Arthrosc* 2024; 32: 1077–86.

8 Butler JJ, Harrington MC, Tong Y, et al. From jargon to clarity: improving the readability of foot and ankle radiology reports with an artificial intelligence large language model. *Foot Ankle Surg* 2024; 30: 331–37.

9 Çamur E, Cesur T, Güneş YC. A comparative study: performance of large language models in simplifying Turkish computed tomography reports. *J Istanb Univ Fac Med* 2024; 87: 321–26.

10 Can E, Uller W, Vogt K, et al. Large language models for simplified interventional radiology reports: a comparative analysis. *Acad Radiol* 2025; 32: 888–98.

11 Cesur YCG, Çamur E. Use of large language models in radiological reports: a study on simplifying Turkish MRI findings. *Ann Clin Anal Med* 2024; 15: 586–90.

12 Chung EM, Zhang SC, Nguyen AT, Atkins KM, Sandler HM, Kamrava M. Feasibility and acceptability of ChatGPT-generated radiology report summaries for cancer patients. *Digit Health* 2023; 9: 20552076231221620.

13 Doshi R, Amin KS, Khosla P, Bajaj SS, Chheang S, Forman HP. Quantitative evaluation of large language models to streamline radiology report impressions: a multimodal retrospective analysis. *Radiology* 2024; 310: e231593.

14 Gupta A, Singh S, Malhotra H, et al. Provision of radiology reports simplified with large language models to patients with cancer: impact on patient satisfaction. *JCO Clin Cancer Inform* 2025; 9: e2400166.

15 Güneş YC, Cesur T, Çamur E. Comparative analysis of large language models in simplifying Turkish ultrasound reports to enhance patient understanding. *Eur J Ther* 2024; 30: 714–23.

16 Jeblick K, Schachtner B, Dexl J, et al. ChatGPT makes medicine easy to swallow: an exploratory case study on simplified radiology reports. *Eur Radiol* 2024; 34: 2817–25.

17 Kuckelman IJ, Wetley K, Yi PH, Ross AB. Translating musculoskeletal radiology reports into patient-friendly summaries using ChatGPT-4. *Skeletal Radiol* 2024; 53: 1621–24.

18 Li H, Moon JT, Iyer D, et al. Decoding radiology reports: potential application of OpenAI ChatGPT to enhance patient understanding of diagnostic reports. *Clin Imaging* 2023; 101: 137–41.

19 Li HH, Moon JT, Kumar S, et al. Evaluation of multilingual simplifications of IR procedural reports using GPT-4. *J Vasc Interv Radiol* 2025; 36: 696–703.e1.

20 Lyu Q, Tan J, Zapadka ME, et al. Translating radiology reports into plain language using ChatGPT and GPT-4 with prompt learning: results, limitations, and potential. *Vis Comput Ind Biomed Art* 2023; 6: 9.

21 Maroncelli R, Rizzo V, Pasculli M, et al. Probing clarity: AI-generated simplified breast imaging reports for enhanced patient comprehension powered by ChatGPT-4o. *Eur Radiol Exp* 2024; 8: 124.

22 Park J, Oh K, Han K, Lee YH. Patient-centered radiology reports with generative artificial intelligence: adding value to radiology reporting. *Sci Rep* 2024; 14: 13218.

23 Pisarcik D, Kissling M, Heimer J, et al. Artificial intelligence language models to translate professional radiology mammography reports into plain language—impact on interpretability and perception by patients. *Acad Radiol* 2025; 32: 4988–96.

24 Prucker P, Busch F, Dorfner F, et al. Performance of open-source and proprietary large language models in generating patient-friendly radiology chest CT reports. *Clin Imaging* 2025; 125: 110557.

25 Rogasch JMM, Metzger G, Preisler M, et al. ChatGPT: can you prepare my patients for [18F]FDG PET/CT and explain my reports? *J Nucl Med* 2023; 64: 1876–79.

26 Salam B, Kravchenko D, Nowak S, et al. Generative Pre-trained Transformer 4 makes cardiovascular magnetic resonance reports easy to understand. *J Cardiovasc Magn Reson* 2024; 26: 101035.

27 Sarangi PK, Lumbani A, Swarup MS, et al. Assessing ChatGPT's proficiency in simplifying radiological reports for healthcare professionals and patients. *Cureus* 2023; 15: e50881.

28 Schmidt S, Zimmerer A, Cucos T, Feucht M, Navas L. Simplifying radiologic reports with natural language processing: a novel approach using ChatGPT in enhancing patient understanding of MRI results. *Arch Orthop Trauma Surg* 2024; 144: 611–18.

29 Stephan D, Bertsch AS, Schumacher S, et al. Improving patient communication by simplifying AI-generated dental radiology reports with ChatGPT: comparative study. *J Med Internet Res* 2025; 27: e73337.
30 Sterling NW, Brann F, Frisch SO, Schrager JD. Patient-readable radiology report summaries generated via large language model: safety and quality. *J Patient Exp* 2024; 11: 23743735241259477.
31 Sudarshan M, Shih S, Yee E, et al. Agentic LLM workflows for generating patient-friendly medical reports. *arXiv* 2024; published online Aug 2. http://arxiv.org/abs/2408.01112 (preprint).
32 Sunshine A, Honce GH, Callen AL, et al. Evaluating the quality and understandability of radiology report summaries generated by ChatGPT: survey study. *JMIR Form Res* 2025; 9: e76097–76097.
33 Tang CC, Nagesh S, Fussell DA, et al. Generating colloquial radiology reports with large language models. *J Am Med Inform Assoc* 2024; 31: 2660–67.
34 Tariq A, Trivedi S, Urooj A, et al. Patient-centric summarization of radiology findings using two-step training of large language models. *ACM Trans Comput Healthc* 2025; 6: 21.
35 Tepe M, Emekli E. Decoding medical jargon: the use of AI language models (ChatGPT-4, BARD, Microsoft Copilot) in radiology reports. *Patient Educ Couns* 2024; 126: 108307.
36 Tripathi S, Mutter L, Muppuri M, et al. PRECISE framework: enhanced radiology reporting with GPT for improved readability, reliability, and patient-centered care. *Eur J Radiol* 2025; 187: 112124.
37 van Driel MHE, Blok N, van den Brand JAJG, et al. Leveraging GPT-4 enables patient comprehension of radiology reports. *Eur J Radiol* 2025; 187: 112111.
38 Yang Z, Cherian S, Vucetic S. Two-pronged human evaluation of ChatGPT self-correction in radiology report simplification. *Find ACL* 2024; 2024: 4701–14.

References of the discussion section

70 Amin K, Khosla P, Doshi R, Chheang S, Forman HP. Artificial intelligence to improve patient understanding of radiology reports. Yale J Biol Med 2023; 96: 407–17.

71 Shah SJ, Nair A, Murtagh K, et al. Clinician Perspectives on AI-Generated Drafts of Patient Test Result Explanations. JAMA Netw Open 2025; 8: e2528794.

72 Artsi Y, Klang E, Collins JD, et al. Large language models in radiology reporting - A systematic review of performance, limitations, and clinical implications. Intell Based Med 2025; 12: 100287.

73 Wenderott K, Krups J, Weigl M, Wooldridge AR. Facilitators and Barriers to Implementing AI in Routine Medical Imaging: Systematic Review and Qualitative Analysis. J Med Internet Res 2025; 27: e63649.

74 Rotholz S, Lin C-T. 'I don't think it should take you three days to tell me my baby is dead.' A case of fetal demise: unintended consequences of immediate release of information. J Am Med Inform Assoc 2023; 30: 1301–4.

75 Steitz BD, Turer RW, Lin C-T, et al. Perspectives of patients about immediate access to test results through an online patient portal. JAMA Netw Open 2023; 6: e233572.

76 Bedi S, Liu Y, Orr-Ewing L, et al. Testing and Evaluation of Health Care Applications of Large Language Models: A Systematic Review. JAMA 2025; 333: 319–28.

77 Reichenpfader D, Müller H, Denecke K. A scoping review of large language model based approaches for information extraction from radiology reports. NPJ Digit Med 2024; 7: 222.

78 Lopez C, Kim B, Sacks K. Health literacy in the United States: Enhancing assessments and reducing disparities. SSRN Electron J 2022; published online May 20. DOI:10.2139/ssrn.4182046.

79 Lee H-S, Song S-H, Park C, et al. The ethics of simplification: balancing patient autonomy, comprehension, and accuracy in AI-generated radiology reports. BMC Med Ethics 2025; 26: 136.

80 Lee H-S, Kim S, Kim S, et al. Readability versus accuracy in LLM-transformed radiology reports: stakeholder preferences across reading grade levels. Radiol Med 2025; published online Sept 29. DOI:10.1007/s11547-025-02098-5.

81 Tanprasert T, Kauchak D. Flesch-Kincaid is not a text simplification evaluation metric. In: Proceedings of the 1st Workshop on

Natural Language Generation, Evaluation, and Metrics (GEM 2021). Stroudsburg, PA, USA: Association for Computational Linguistics, 2021: 1–14.

82 Perlis N, Finelli A, Lovas M, et al. Creating patient-centered radiology reports to empower patients undergoing prostate magnetic resonance imaging. Can Urol Assoc J 2021; 15: 108–13.

83 Alarifi M, Patrick T, Jabour A, Wu M, Luo J. Designing a consumer-friendly radiology report using a patient-centered approach. J Digit Imaging 2021; 34: 705–16.

84 Zhang Z, Citardi D, Wang D, Genc Y, Shan J, Fan X. Patients' perceptions of using artificial intelligence (AI)-based technology to comprehend radiology imaging data. Health Informatics J 2021; 27: 14604582211011215.

85 Lockwood P, Mitchell M. A co-designed patient reported experience measure for understanding the patient's and public experience of receiving X-ray results. Radiography (Lond) 2025; 31: 102990.

86 Grover V, Balusamy B, M. K. N, Anand V, Milanova M. Approaches to Human-Centered AI in Healthcare. IGI Global, 2024.

87 Herwald SE, Shah P, Johnston A, Olsen C, Delbrouck J-B, Langlotz CP. RadGPT: A System Based on a Large Language Model That Generates Sets of Patient-Centered Materials to Explain Radiology Report Information. J Am Coll Radiol 2025; published online June 10. DOI:10.1016/j.jacr.2025.06.013.

88 Prinz A, Golke S, Wittwer J. How accurately can learners discriminate their comprehension of texts? A comprehensive meta-analysis on relative metacomprehension accuracy and influencing factors. Educ Res Rev 2020; 31: 100358.

89 Aydin S, Karabacak M, Vlachos V, Margetis K. Large language models in patient education: a scoping review of applications in medicine. Front Med (Lausanne) 2024; 11: 1477898.

90 Rockall AG, Justich C, Helbich T, Vilgrain V. Patient communication in radiology: Moving up the agenda. Eur J Radiol 2022; 155: 110464.

91 Schreyer AG, Schneider K, Dendl LM, et al. Patient Centered Radiology - An Introduction in Form of a Narrative Review. Rofo 2022; 194: 873–81.

92 Mityul MI, Gilcrease-Garcia B, Mangano MD, Demertzis JL, Gunn AJ. Radiology reporting: Current practices and an introduction to patient-centered opportunities for improvement. AJR Am J Roentgenol 2017; 210: 1–10.

Use of ChatGPT in the manuscript

During the preparation of this work the authors used ChatGPT Business (GPT-5, OpenAI; data not used for training) on Sept 1, 2025, to generate a draft for the research in context panel based on the abstract using the following prompt:

The prompt used was "you are an expert medical writer. Write a draft of the Lancet Digital Health research in context panel based on my abstract ">>abstract<<" ? Follow the following journal guidance: Panel: Research in context Evidence before this study This section should include a description of all the evidence that the authors considered before undertaking the systematic review, in order for the reader to understand the implications of this study in the context of other similar previously available research. Authors should briefly state: the sources (databases, journal or book reference lists, etc) searched; the criteria used to include or exclude studies (including the exact start and end dates of the search), which should not be limited to English language publications; the search terms used; the quality (risk of bias) of that evidence; and the pooled estimate derived from meta-analysis of the evidence, if appropriate. Added value of this study Authors should describe here how their findings add value to the existing evidence. Implications of all the available evidence Authors should state the implications for practice or policy and future research of their study combined with existing evidence."
